# Hepatic Mechanisms and Therapeutic Target Underpinning Hypercoagulation in Obesity: Insights From Genetic and Transcriptomic Analyses

**DOI:** 10.1002/exp2.70210

**Published:** 2026-08-13

**Authors:** Shumin Li, Gengchen Feng, Xin Huang, Xiao Teng, Ziyi Yang, Yue Liu, Yonghui Jiang, Tao Zhu, Teng Liu, Yunfei Xu, Chang Pan, Han Zhao, Shaozhuang Liu, Shigang Zhao, Zi‐Jiang Chen

**Affiliations:** ^1^ State Key Laboratory of Reproductive Medicine and Offspring Health Center for Reproductive Medicine Institute of Women Children and Reproductive Health Shandong University Jinan China; ^2^ National Research Center for Assisted Reproductive Technology and Reproductive Genetics Shandong University Jinan Shandong China; ^3^ Key Laboratory of Reproductive Endocrinology Ministry of Education Shandong University Jinan Shandong China; ^4^ Division of Bariatric and Metabolic Surgery Department of General Surgery Qilu Hospital of Shandong University Jinan Shandong China; ^5^ The Second Hospital of Shandong University Jinan Shandong China; ^6^ Department of Obstetrics and Gynecology Qilu Hospital of Shandong University Jinan Shandong China; ^7^ Department of General Surgery Qilu Hospital of Shandong University Jinan Shandong China; ^8^ Department of Emergency Qilu Hospital Cheeloo College of Medicine Shandong University Jinan Shandong China; ^9^ Shandong Technology Innovation Center for Reproductive Health Jinan Shandong China; ^10^ Shandong Provincial Clinical Research Center for Reproductive Health Jinan Shandong China; ^11^ Shandong Key Laboratory of Reproductive Research and Birth Defect Prevention Jinan Shandong China; ^12^ Research Unit of Gametogenesis and Health of ART‐Offspring Chinese Academy of Medical Sciences & Peking Union Medical College (No. 2021RU001) Jinan Shandong China

**Keywords:** fibrinogen, genetic analysis, hepatic transcriptomics, hypercoagulation, obesity

## Abstract

Obesity predisposes individuals to hypercoagulation, a key factor in life‐threatening complications like stroke and cardiovascular disease. While clotting factors are primarily secreted by the liver, the mechanisms linking obesity to hypercoagulation remain unclear. In this study, we established an obesity cohort and conducted genetic analyses, single‐nucleus and bulk RNA sequencing of human liver tissues, and in vitro experiments to elucidate the underlying mechanisms. Genetic analysis identified a causal relationship between body mass index (BMI) and coagulation parameters, specifically elevated fibrinogen levels. Clinical cohort data demonstrated a significant association between BMI and plasma fibrinogen levels and fibrinogen function. Hepatic transcriptomic data revealed a significant link between BMI and hepatic fibrinogen expression. snRNA‐seq data and deconvolution analysis of bulk RNA‐seq uncovered a specific hepatocyte subpopulation (Hep5) that was significantly increased and responsible for aberrant coagulation factor secretion in obesity. Pseudotime trajectory analysis indicated that increased interleukin 6 (IL6) signaling in obese patients contributed to the activation of the hepatocyte identity‐related transcription factor CEBPB, promoting the differentiation of other hepatocyte subpopulations into Hep5. Further analysis in an independent obesity cohort confirmed serum IL6 as a critical mediator of the BMI‐fibrinogen relationship, supporting the potential value of IL6 antibodies, which are currently in Phase 2b trials, for improving coagulation abnormalities. In vitro experiments demonstrated that the CEBPB inhibitor helenalin acetate reversed the aberrant expression of hepatocyte differentiation‐related genes induced by IL6. This study defines the hepatic transcriptomic landscape in obesity, elucidates mechanisms of fibrinogen elevation, and highlights therapeutic targets for managing hypercoagulation in obesity.

## Graphical abstract

Obesity‐linked hypercoagulation is driven by fibrinogen overproduction in a specific hepatocyte cluster via IL6 signalling. Genetic and mechanistic studies reveal this pathway as a key target for therapies to mitigate clotting risks and associated complications in obese individuals.

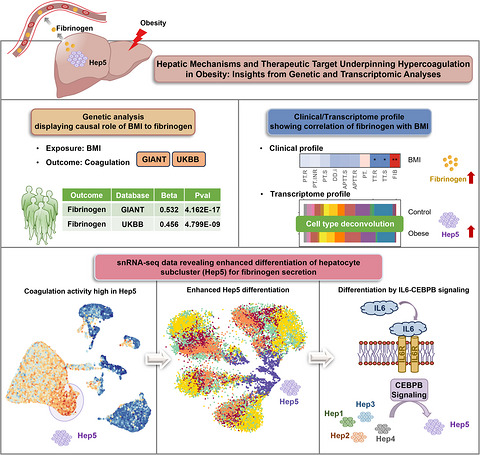

**Abbreviations**
APTT: activated partial thromboplastin time
CEBPB: CCAAT enhancer binding protein beta
CRP: C‐reactive protein
CV: central venous
FIB: fibrinogen
HA: helenalin acetate
IL6: interleukin 6
IL6R: interleukin 6 receptor
MASLD: metabolic dysfunction‐associated steatotic liver disease
PP: periportal
PPI: protein–protein interaction
PT: prothrombin time
TF: transcription factor
TT: thrombin time

## 1 Introduction

Obesity represents a significant health challenge, with approximately 650 million adults (out of 5.5 billion people) diagnosed with obesity, globally [1]. The prevalence of obesity varies between countries and genders, ranging from 1.7% to 65.3%, steadily rising each year [1, 2, 3]. Evidence from clinical and epidemiological studies has clearly established coagulation disorder as a common feature of obesity [4] associated with potentially severe adverse outcomes, such as ischemic stroke, myocardial infarction and pulmonary embolism [5, 6]. Obese individuals have several elevated risk factors leading to coagulation disorders, including platelet hyperactivity, decreased fibrinolysis [7, 8] and abnormal secretion of adipokines and proinflammatory factors [9, 10], such as elevated IL6, TNFα and IL1. However, studies of obesity and hypercoagulability have generally focused on clinical findings while mechanistic details are lacking.

As the largest parenchymal organ in humans, the liver exhibits significant heterogeneity, primarily comprising a variety of hepatocyte types distributed across different anatomical regions and performing diverse functions [11, 12, 13]. The liver is responsible for essential functions that include participating in nutrient metabolism, synthesizing circulating proteins, and contributing to coagulation processes [14, 15]. To mediate the latter function, the liver secretes numerous coagulation factors, especially fibrinogen and prothrombin, as well as fibrinolytic enzymes [16]. During both acute and chronic liver injury, the liver initiates repair and regeneration processes in which mature hepatocytes or liver stem cells differentiate to replenish and restore hepatocyte function [17, 18]. Although obesity is associated with hepatocyte damage and numerous liver diseases, such as metabolic dysfunction‐associated steatotic liver disease (MASLD), liver fibrosis, and hepatocellular carcinoma (HCC) [10, 19, 20, 21], the mechanisms through which obesity induces abnormalities in coagulation via its effects on hepatocyte function or differentiation remain poorly understood.

In this study, Mendelian randomization analysis identified a significant causal relationship between body mass index (BMI) and elevated fibrinogen levels. To investigate the hepatic mechanisms underlying this obesity‐related elevation of fibrinogen, we collected liver biopsy samples from obese individuals undergoing sleeve gastrectomy for morbid obesity and non‐obese control patients undergoing surgery for a benign liver tumor. Integration of clinical profiles with bulk and single‐nucleus (sn) RNA‐seq of these samples revealed a specific hepatocyte subpopulation (Hep5) responsible for secreting coagulation factors that was significantly increased in the livers of obese individuals, potentially through IL6‐CEBPB‐induced stimulation of differentiation pathways in other hepatocyte cell types. This study depicts the transcriptomic landscape in the liver of obese individuals, and partially elucidates the hepatic mechanisms contributing to the obesity‐related hypercoagulable state, cumulatively offering a valuable resource for future research and new therapeutic strategies for hypercoagulation in obesity.

## 2 Results

### 2.1 Mendelian Randomization Shows BMI is a Causal Factor in Elevated Fibrinogen Levels

We selected instrumental variables (IVs) following criteria described in the Methods section. Details of all SNPs related to the BMI trait and coagulation traits obtained from the Genetic Investigation of ANthropometric Traits (GIANT) consortium and UK Biobank (UKBB) for use in the Mendelian randomization (MR) analysis are summarized in Table S1, Supporting Information. Coagulation parameters included 13 coagulation factors, as well as plasminogen, plasmin, mean platelet volume, platelet count, platelet distribution width and *d*‐dimer measurements. The *F*‐statistics of all genetic IVs were higher than 10, indicating good strength. We systematically screened and excluded SNPs associated with known confounding traits—such as smoking (e.g., rs4660443), alcohol consumption (e.g., rs1064213), type 2 diabetes (e.g., rs2235564), inflammatory markers, as well as liver and kidney diseases. The MR analysis revealed significant associations between BMI and elevated fibrinogen (*P* = 4.162 × 10^−17^ in GIANT and *P* = 4.799 × 10^−9^ in UKBB) and reduced calcium ion (*P* = 1.635 × 10^−5^ in GIANT) after Bonferroni correction for multiple testing (*p* < 0.05/42). See Table 1 for complete results.

**TABLE 1:** Mendelian randomization analysis of the causal effects of BMI on coagulation parameter.

Outcome	Database	nSNP	Beta	Beta (95%CI)	pval
Fibrinogen	GIANT	34	0.532	0.532 (0.408, 0.656)	4.162E‐17
Fibrinogen	UKBB	16	0.456	0.456 (0.303, 0.609)	4.799E‐09
Prothrombin	GIANT	173	−0.011	−0.011 (−0.182, 0.161)	0.900
Prothrombin	UKBB	100	−0.147	−0.147 (−0.326, 0.032)	0.107
Thrombin	GIANT	172	−0.083	−0.083 (−0.256, 0.09)	0.348
Thrombin	UKBB	100	−0.086	−0.086 (−0.26, 0.088)	0.335
Tissue Factor	GIANT	172	0.098	0.098 (−0.04, 0.235)	0.163
Tissue Factor	UKBB	100	0.041	0.041 (−0.1, 0.182)	0.571
Calcium ion	GIANT	159	−0.068	−0.068 (−0.098, ‐0.037)	1.635E‐05
Calcium ion	UKBB	99	0.017	0.017 (−0.035, 0.07)	0.518
Coagulation Factor V	GIANT	171	−0.002	−0.002 (−0.183, 0.178)	0.979
Coagulation Factor V	UKBB	100	0.025	0.025 (−0.155, 0.205)	0.784
Coagulation Factor VII	GIANT	173	0.051	0.051 (−0.116, 0.217)	0.551
Coagulation Factor VII	UKBB	99	−0.014	−0.014 (−0.189, 0.162)	0.879
Coagulation Factor VIII	GIANT	173	0.005	0.005 (−0.158, 0.167)	0.954
Coagulation Factor VIII	UKBB	100	−0.026	−0.026 (−0.214, 0.162)	0.785
Coagulation Factor IX	GIANT	173	0.255	0.255 (0.092, 0.417)	0.002
Coagulation Factor IX	UKBB	100	0.102	0.102 (−0.086, 0.29)	0.286
Coagulation Factor IXab	GIANT	173	0.247	0.247 (0.085, 0.408)	0.003
Coagulation Factor IXab	UKBB	100	0.085	0.085 (−0.092, 0.261)	0.347
Coagulation Factor X	GIANT	173	−0.001	−0.001 (−0.164, 0.163)	0.994
Coagulation Factor X	UKBB	99	−0.079	−0.079 (−0.27, 0.112)	0.420
Coagulation Factor Xa	GIANT	173	0.009	0.009 (−0.155, 0.173)	0.911
Coagulation Factor Xa	UKBB	100	−0.045	−0.045 (−0.224, 0.134)	0.619
Coagulation Factor XI	GIANT	173	−0.057	−0.057 (−0.23, 0.116)	0.518
Coagulation Factor XI	UKBB	98	0.055	0.055 (−0.145, 0.255)	0.591
Coagulation Factor XIII	GIANT	169	−0.012	−0.012 (−0.184, 0.16)	0.889
Coagulation Factor XIII	UKBB	100	−0.176	−0.176 (−0.349, ‐0.003)	0.046
Coagulation Factor XIII B chain	GIANT	173	0.025	0.025 (−0.145, 0.196)	0.771
Coagulation Factor XIII B chain	UKBB	100	−0.193	−0.193 (−0.37, ‐0.017)	0.031
Plasminogen	GIANT	173	−0.051	−0.051 (−0.213, 0.112)	0.540
Plasminogen	UKBB	99	0.166	0.166 (−0.026, 0.357)	0.090
Plasmin	GIANT	172	0.039	0.039 (−0.27, 0.349)	0.803
Plasmin	UKBB	100	0.284	0.284 (−0.049, 0.618)	0.095
Platelet count	GIANT	162	−0.029	−0.029 (−0.065, 0.008)	0.123
Platelet count	UKBB	87	−0.004	−0.004 (−0.038, 0.03)	0.823
Mean platelet volume	GIANT	165	0.006	0.006 (−0.025, 0.038)	0.696
Mean platelet volume	UKBB	87	0.006	0.006 (−0.024, 0.036)	0.704
Platelet distribution width	GIANT	171	0.006	0.006 (−0.028, 0.041)	0.718
Platelet distribution width	UKBB	96	−0.001	−0.001 (−0.042, 0.04)	0.963
D‐dimer	GIANT	171	0.065	0.065 (−0.106, 0.237)	0.456
D‐dimer	UKBB	100	−0.016	−0.016 (−0.192, 0.16)	0.858

^*^The results were generated by Inverse Variance Weighting (IVW) approach. Significant associations were identified with *P* value < 0.05/42, after applying Bonferroni correction for multiple testing. Red represents the increase in coagulation factor levels with increasing BMI; blue represents the decrease in coagulation factor levels with increasing BMI.

### 2.2 Clinical Profiles Support the Causal Relationship Between BMI and Fibrinogen Levels

**FIGURE 1:**
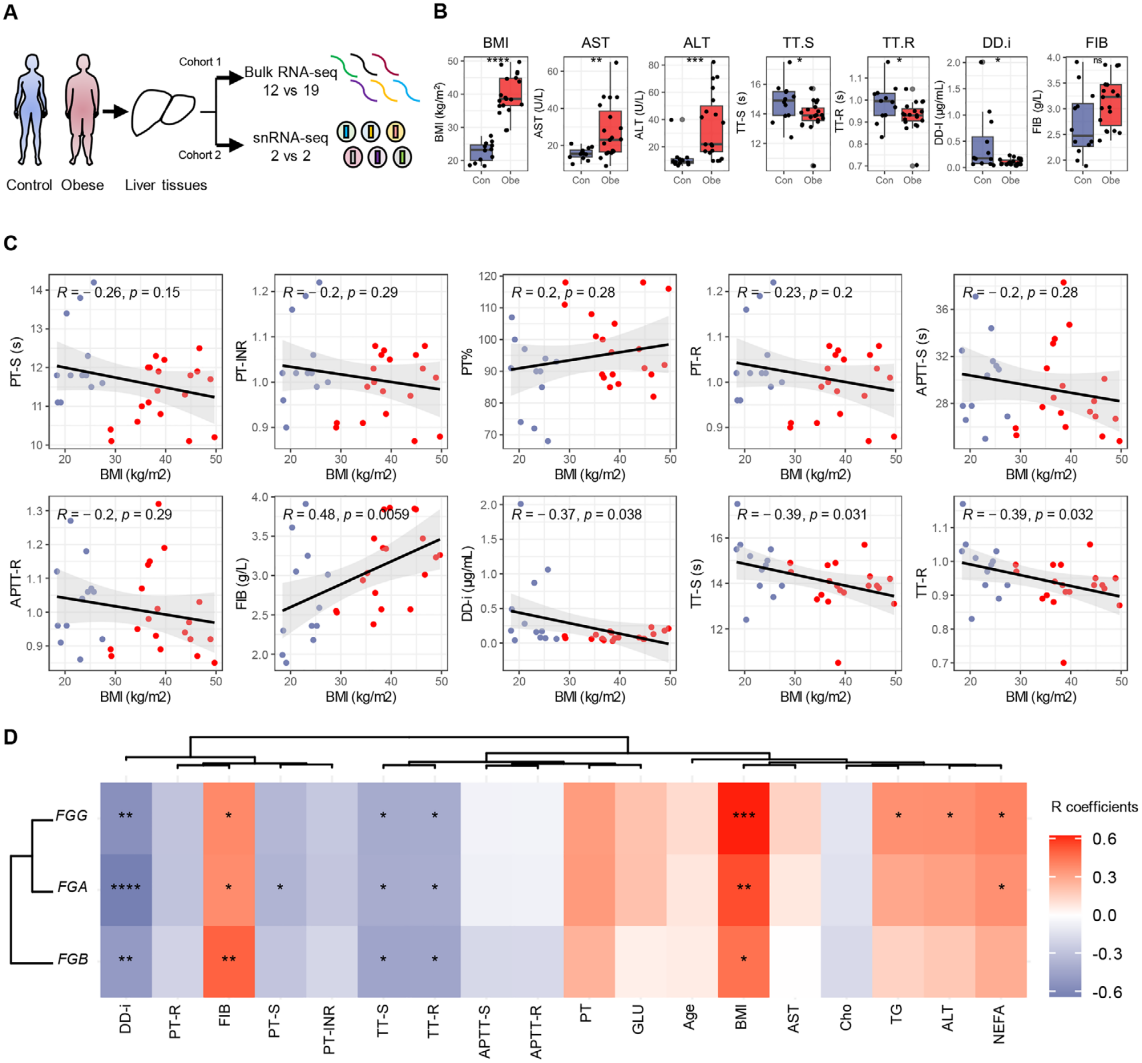
Increased BMI leads to elevated plasma fibrinogen and hepatic fibrinogen expression. (A) Study design of two cohorts for bulk RNA‐seq (Cohort 1: control vs. obese = 12 vs. 19) and for snRNA‐seq (control vs. obese = 2 vs. 2) separately. Con, control group; Obe, obese group. (B) Clinical characteristics of individuals in Cohort 1 compared between the obese and control groups. Student's *t*‐test. *p < 0.05; **p < 0.01; ***p < 0.001. Body mass index (BMI); aspartate aminotransferase (AST); alanine aminotransferase (ALT); thrombin time (screening) (TT‐S); thrombin time (ratio) (TT‐R); D‐dimer (DD‐i); fibrinogen (FIB). (C) Relationship between BMI and coagulation‐related parameters in Cohort 1. Purple dots represent the control group (*n* = 12), and red dots represent the Obese group (*n* = 19). Correlation and *p*‐values (plotted black line with 95% confidence interval) are indicated. (D) Heatmap showing the Spearman correlations of fibrinogen genes (*FGA*, *FGB* and *FGG*) mRNA expression detected in bulk RNA‐seq with different clinical characteristics of patients in Cohort 1. Significant correlations with adjusted *p*‐values < 0.05 are labelled in the heatmap with asterisks. *adjusted *p* < 0.05; **adjusted *p* < 0.01; ***adjusted *p* < 0.001.

Considering that most coagulation factors are synthesized in the liver, we next sought to determine how obesity promotes hypercoagulability through hepatic effects by employing two distinct approaches, that is, bulk and snRNA‐seq, to characterize liver‐associated transcriptomic features in obese individuals. Liver tissues for the control group (Con) were obtained from healthy liver regions of normal‐weight individuals who underwent partial hepatectomy for benign liver tumors, while obese group (Obe) liver tissues were obtained from individuals who underwent sleeve gastrectomy for morbid obesity. Liver tissues from Cohort 1 were collected for bulk RNA‐seq (Figure 1A, Con vs. Obe = 12 vs. 19), while concurrently, snRNA‐seq was performed using frozen liver tissues from Cohort 2, including two control individuals and two obese individuals (Figure 1A).

Obese individuals were defined by a BMI greater than 28 kg m^−2^, with exclusion criteria including history of diabetes mellitus, severe hepatic dysfunction and/or prior use of metabolic medications [22]. Examination of clinical characteristics of Cohort 1 individuals revealed that BMI and liver transaminase levels (ALT, AST) were significantly elevated in the obese group (Figure 1B). Evaluation of coagulation indicators showed obvious decreases in thrombin time (TT) assays, including TT‐S and TT‐R, as well as D‐dimer levels, in obese participants, with plasma fibrinogen levels showing an increasing trend (Figure 1B). By contrast, the levels of other coagulation indicators, such as prothrombin time (PT) and activated partial thromboplastin time (APTT), were comparable between groups (Figure S1A, Supporting Information). As TT assays reflected both the quantity and functionality of fibrinogen in the body, while PT and APTT assays reflected extrinsic and intrinsic coagulation, respectively, which collectively indicated a predominant hypercoagulable state characterized by enhanced fibrinogen contents or functionality in obese individuals.

Calculation of correlation coefficients between coagulation markers and other clinical indicators indicated that fibrinogen was most associated with BMI (*R* = 0.48, *p* = 0.0059**, Figure 1C; Figure S1B, Supporting Information), whereas TT‐S (*R* = −0.39, *p* = 0.031*), TT‐R (*R* = −0.39, *p* = 0.032*) and D‐dimer (*R* = −0.37, *p* = 0.038*) exhibited negative correlations with BMI (Figure 1C). However, no significant correlations were observed between BMI and PT or APTT parameters.

Moreover, considering the liver serves as the hub of fibrinogen synthesis, we examined possible associations between the clinical parameters and expression levels of fibrinogen‐coding genes *FGA*, *FGB*, and *FGG* mRNA in bulk RNA‐seq data of individuals in Cohort 1. In addition to significant positive correlations between *FGA*, *FGB* and *FGG* mRNA levels and plasma fibrinogen, their expression levels were also significantly positively correlated with BMI (Figure 1D). Together, these results suggested that elevated BMI could indeed function as a driving factor in the increased hepatic synthesis of fibrinogen.

### 2.3 A Single‐Nucleus Atlas of the Liver in Obesity

**FIGURE 2:**
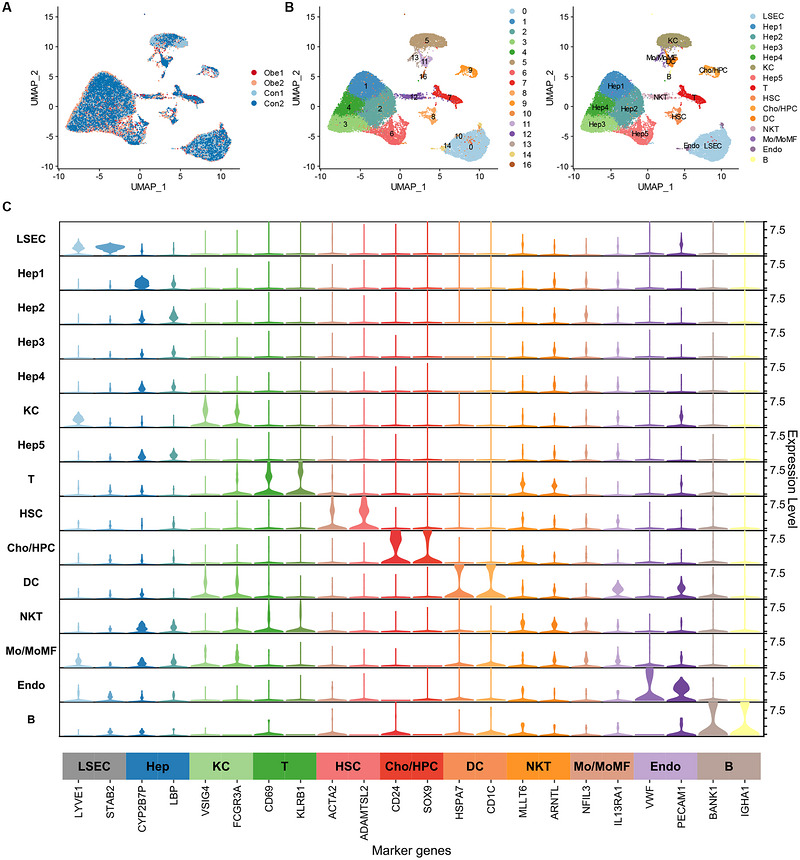
Single nucleus RNA‐seq (snRNA‐seq) landscape of control and obese individual livers. (A) Uniform manifold approximation and projection (UMAP) plot of liver cells sequenced from different individuals. Con1 and Con2 are from the control (Con) group, while Obe1 and Obe2 are from the obese (Obe) group. (B) UMAP plot of liver cell clusters based on 27,376 single‐nucleus transcriptomes. Cell type annotations for each cluster are indicated in the plot (right). These include liver sinusoidal endothelial cells (LSEC), hepatocytes (Hep1, Hep2, Hep3, Hep4, and Hep5), Kupffer cells (KC), T cells (T), hepatic stellate cells (HSC), cholangiocyte/hepatic progenitor cells (Cho/HPC), dendritic cells (DC), natural killer T cells (NKT), monocytes and monocyte‐derived macrophages (Mo/MoMF), endothelial cells (Endo) and B cells (B). (C) Expression of representative enriched genes for each cell type. Gene expression violin plots are shown in log‐scale UMI (scaledata).

Hepatocytes are well‐recognized as the cells predominantly responsible for fibrinogen secretion. To explore whether fibrinogen synthesis was specific to any hepatocyte subclusters, we performed snRNA‐seq for 31,810 total nuclei, which decreased to 27,376 high‐quality nuclei after filtering and quality control. These data included 11,402 nuclei from normal livers and 15,974 nuclei from obese livers, and identified 30,939 total genes (Figure 2A, Figure S1C). UMAP analysis of these single nucleus transcriptomes identified distinct cell clusters after removing one cluster comprised of mixed blood cells (Figure 2B). These clusters were then annotated as specific cell types based on their expression of established marker genes (Figure 2C, Table S2, Supporting Information), including five clusters of hepatocytes (*CYP2B7P*, *LBP*), liver sinusoidal endothelial cells (*LYVE1*, *STAB2*), endothelial cells (*VWF*, *PECAM1*), hepatic stellate cells (*ACTA2*, *ADAMTSL2*), Kupffer cells (*VSIG4*, *FCGR3A*), cholangiocytes/hepatic progenitor cells (*CD24*, *SOX9*), B cells (*BANK1*, *IGHA1*), natural killer T cells (*MLLT6*, *ARNTL*), T cells (*CD69*, *KLRB1*), monocytes and monocyte‐derived macrophages (*NFIL3*, *IL13RA1*) and dendritic cells (*HSPA7*, *CD1C*) (Figure 2B).

### 2.4 An Increased Hep5 Proportion is Responsible for Coagulation Pathway Activity in Obese Liver

**FIGURE 3:**
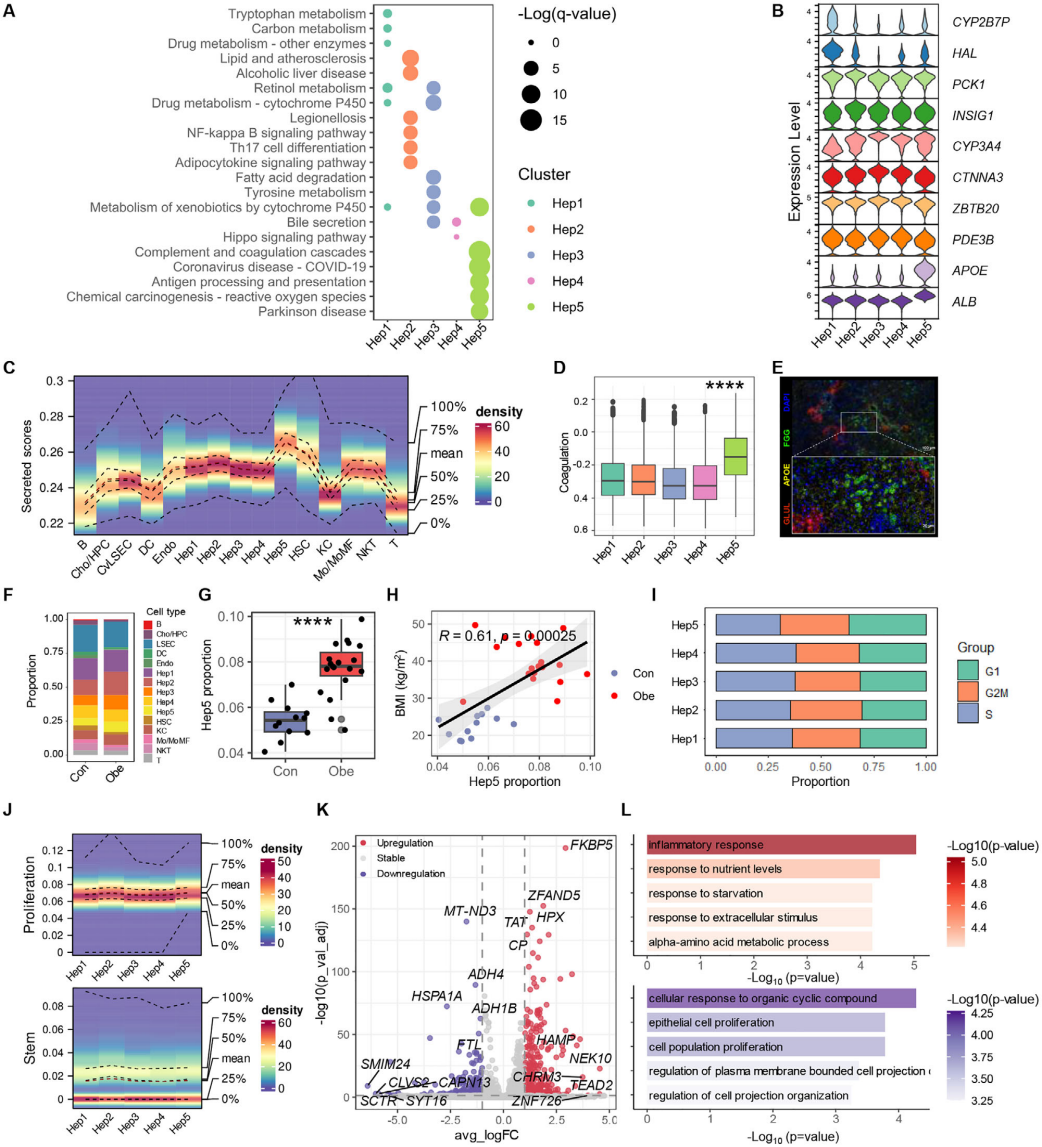
Increased proportion of Hep5 cluster responsible for hypercoagulation in obese individuals. (A) KEGG pathway enrichment analysis of marker genes in each hepatocyte cluster (Hep1, Hep2, Hep3, Hep4, and Hep5). (B) Expression of representative marker genes for each hepatocyte cluster. (C) Density plot of secreted scores across all cell types based on a gene set composed of all secreted proteins according to the Human Protein Atlas (HPA) database. (D) Coagulation activity of each hepatocyte cluster based on the HALLMARK_COAGULATION gene set. ****adjusted *p*‐value < 0.0001 using Kruskal–Wallis test, followed by pairwise comparisons with Dunn's test to control for multiple comparisons. (E) Triple immunofluorescence staining of human liver sections showing: nuclear DAPI (blue), Hep5 markers FGG (green) and APOE (yellow), and CV hepatocyte marker GLUL (red). The co‐localization of FGG and APOE confirms the presence of Hep5 populations, while their spatial relationship to GLUL^+^ cells demonstrates their lobular distribution. (F) Deconvolution result showing the proportion of each cell type in Cohort 1 according to bulk RNA‐seq data. (G) Boxplot showing the significantly increased proportion of Hep5 in obese (Obe) individuals compared to control (Con) individuals in Cohort 1. Student's *t*‐test, ****P < 0.0001. (H) Relationship between BMI and Hep5 proportion in Cohort 1 according to Figure 3F. Purple dots represent the Con group (*n* = 12) and red dots represent the Obe group (*n* = 19). Pearson correlation and adjusted *p*‐values (plotted as a black line with 95% confidence interval) are indicated. (I) Proportion of cells in various cell cycle phases (G1 phase, S phase, and G2/M phase) across five hepatocyte clusters. The cell cycle phase of each cluster is inferred using the *CellCycleScoring* function in Seurat. (J) Density plot of proliferation and stemness scores of each hepatocyte cluster based on BENPORATH_PROLIFERATION.v2023.2.Hs.gmt and CONRAD_STEM_CELL.v2023.2.Hs.gmt in C2 curated gene sets in the MSigDB database. (K) Volcano plot of differential expression analysis of protein‐coding genes in the Hep5 cluster between Obe and Con groups. Genes with |log_2_(fold change)| (log_2_FC) > 1 and adjusted *p*‐value < 0.05 are considered differentially expressed genes (DEGs). Up‐regulated DEGs are colored red, while down‐regulated DEGs are colored purple. The most variable DEGs, with either the smallest adjusted *p*‐value or the largest absolute value of log_2_FC, are labeled with gene names. (L) Pathway enrichment results from Gene Ontology Biological Processes (GOBP) of the upregulated and downregulated DEGs in Figure 3K using the Metascape online tool (https://metascape.org/gp/index.html#/main/step1). Upper panel: up‐regulated DEGs; lower panel: down‐regulated DEGs.

To investigate cell populations responsible for secreting coagulation‐related proteins, we focused on the five hepatocyte subpopulations (Hep1, Hep2, Hep3, Hep4, Hep5). KEGG pathway enrichment and GO functional annotation analyses showed broad heterogeneity in predicted function among these clusters (Figure 3A), with Hep5 exhibiting the most distinct potential functionality, such as complement and coagulation cascades, and antigen processing and presentation, but showing less likely involvement in nutrient metabolism. Quantitative assessment of marker gene transcript levels in snRNA‐seq data for these five hepatocyte clusters revealed significant differential upregulation of several key secretory proteins, such as *APOE* and *ALB*, in Hep5 (Figure 3B).

Closer scrutiny of their spatial distribution across the human liver using publicly available spatial transcriptomic data (GSE185477 and GSE243981) showed that these five hepatocyte clusters had a relatively uniform distribution (Figure S2A–H, Supporting Information).

Further evaluation of secreted protein mRNA expression among different cell clusters, in conjunction with the secreted protein profile defined in the Human Protein Atlas [23] (Table S3, Supporting Information), indicated that the Hep5 cluster displayed the highest expression of secreted proteins, followed by HSCs (Figure 3C). Then, to focus on coagulation pathway activity, we calculated activity scores using single‐sample gene set enrichment analysis (ssGSEA) based on the HALLMARK_COAGULATION gene set. This analysis further supported the likelihood that Hep5 exhibited the highest coagulation activity (Figure 3D). These collective results indicated that the Hep5 hepatocyte subpopulation was primarily responsible for coagulation functions and might indicate that the activity of this cell cluster could potentially share a relationship with BMI in obesity.

To validate our findings of a Hep5 subpopulation that is enriched in coagulation‐related transcripts, such as fibrinogen, we analyzed two additional datasets (GSE185477 and GSE180665) from other studies [24, 25]. Reanalysis and re‐clustering of scRNA‐seq data from healthy human liver (GSE185477) identified six groups, labeled 0–5 (Figure S3A–C, Supporting Information). We found that *FGA* and *FGG* were signature genes of clusters 2 and 4, respectively, and that cluster 4 was enriched in complement and coagulation cascades, while both clusters were enriched in platelet degranulation signature genes. These results illustrated the presence of distinct hepatocyte populations with primary functions related to coagulation. The same analyses in published scRNA‐seq data from healthy human liver (GSE180665) identified four hepatocyte clusters, labeled 0–3 (Figure S3D–F, Supporting Information). Among them, fibrinogen‐related transcripts (*FGA*, *FGB*, and *FGG*) were all signature genes of cluster 2, while other signatures of cluster 2 were enriched in complement and coagulation cascades. These findings support the presence of specialized hepatocyte populations involved in coagulation, consistent with our original observations.

To precisely validate the presence of Hep5 and map Hep5 localization within the hepatic lobular architecture, we conducted triple immunofluorescence co‐staining for FGG (a Hep5 marker), APOE (another Hep5 marker), and GLUL (a central venous hepatocyte marker) on human liver sections. This multicolor imaging analysis not only confirmed the existence of Hep5 populations (APOE^+^FGG^+^ double‐positive hepatocytes) but also demonstrated their distinctive spatial patterning—showing neither preferential periportal nor strict pericentral localization, but rather exhibiting a characteristic interzonal distribution within the hepatic lobule structure (Figure 3E).

When further evaluating the transcriptomic characteristics of Hep5 cells under obesity‐induced stress, gene set enrichment analysis (GSEA) revealed that the ‘complement and coagulation pathways’ were significantly upregulated (Figure S2H, Supporting Information). These findings underscore the activation of coagulation‐related processes within Hep5 cells, suggesting that obesity‐induced stress leads to an expansion of the Hep5 hepatocyte population, a key cell cluster involved in coagulation, while concurrently enhancing their coagulation‐related functional activity.

Subsequently, deconvolution of our tissue‐level bulk RNA‐seq data using our single‐nucleus maps by BisqueRNA (Figure 3F), showed that we primarily captured high‐abundance cell types, as expected (Figure 3G; Table S4 and Figure S4A, Supporting Information). Using these tissue‐deconvolution results to characterize changes in cell‐type proportions revealed that the Hep5 proportion was obviously increased in the obese group (Figure 3G; Figure S4B, Supporting Information). Additionally, another deconvolution method, CIBERSORT, yielded comparable results (Figure S4A,B and Table S4, Supporting Information), and correlation analysis indicated that the proportions of Hep5 and Hep2 populations were significantly positively correlated with BMI in individuals (Figure 3H; Figure S4C, Supporting Information). In particular, Hep5 shared a remarkably high correlation coefficient (0.61) with BMI (*p*‐value = 0.00025). These findings indicated that obesity had the strongest influence on Hep5 cell abundance among the hepatocyte clusters.

Considering the previous MR analysis revealed that BMI is a genetic determinant of increased fibrinogen levels, we subsequently evaluated their expression across hepatocyte clusters. Differential expression analysis of genes related to fibrinogen across different hepatocyte clusters indicated that fibrinogen‐related genes (*FGA*, *FGB*, and *FGG*) were upregulated exclusively in Hep5 cells of obese individuals compared to controls, with no such changes detected in Hep1‐4 cells (Figure S5A, Supporting Information). These observations further support a key role of Hep5 cells in the excessive secretion of fibrinogen in the context of obesity.

To further address potential metabolic confounding, we performed subgroup analyses stratified by MASLD severity (mild vs. severe) and lipid profiles (presence/absence of dyslipidemia) (Figure S5B,C, Supporting Information). Notably, no significant differences in Hep5 cell proportions and fibrinogen‐related genes (*FGA*, *FGB*, and *FGG*) expression were observed across these subgroups. Moreover, we performed additional analyses adjusting for MASLD severity and dyslipidemia status using ANOVA when comparing cell proportions between obese and control groups. Notably, among all hepatocyte subpopulations, only Hep5 maintained a significant between‐group difference after these adjustments (*p* = 0.0059). This suggests that the association between obesity and fibrinogen hypersecretion is independent of MASLD severity or lipid metabolic disturbances, reinforcing the pivotal role of Hep5 cells in mediating this effect.

To uncover the cellular molecular mechanisms leading to increased abundance of Hep5, we first assessed cell cycle state in the hepatocyte cluster transcriptomes. We found that Hep5 cells were predominantly in G1 phase, while relatively few were in S and G2/M phases (Figure 3I). Furthermore, the expression of proliferation‐ and stemness‐related gene sets (Figure 3J) was stable in the Hep5 cluster compared with the other four hepatocyte clusters. Screening for Hep5‐specific signature genes indicated that the transcriptomic profile of Hep5 cells distinctly differed between obese and control individuals, and identified 647 significant differentially expressed protein‐coding genes (DEGs), including 289 up‐ and 358 down‐regulated DEGs between groups (Figure 3K; *P*_adj._ < 0.05, |log_2_FC| > 1, see Table S5, Supporting Information, for DEG list). The most highly enriched pathways in the obese group Hep5 cells included ‘inflammatory response’ and ‘response to nutrient levels’ (Figure 3L, upper), whereas several genes involved in ‘cellular response to organic cyclic compound’ and ‘epithelial cell proliferation’ were down‐regulated (Figure 3L, lower), suggesting that the increase in Hep5 populations was not due to enhanced proliferation.

### 2.5 Hep5 Population Increases Through Enhanced Differentiation of Other Hepatocytes

**FIGURE 4:**
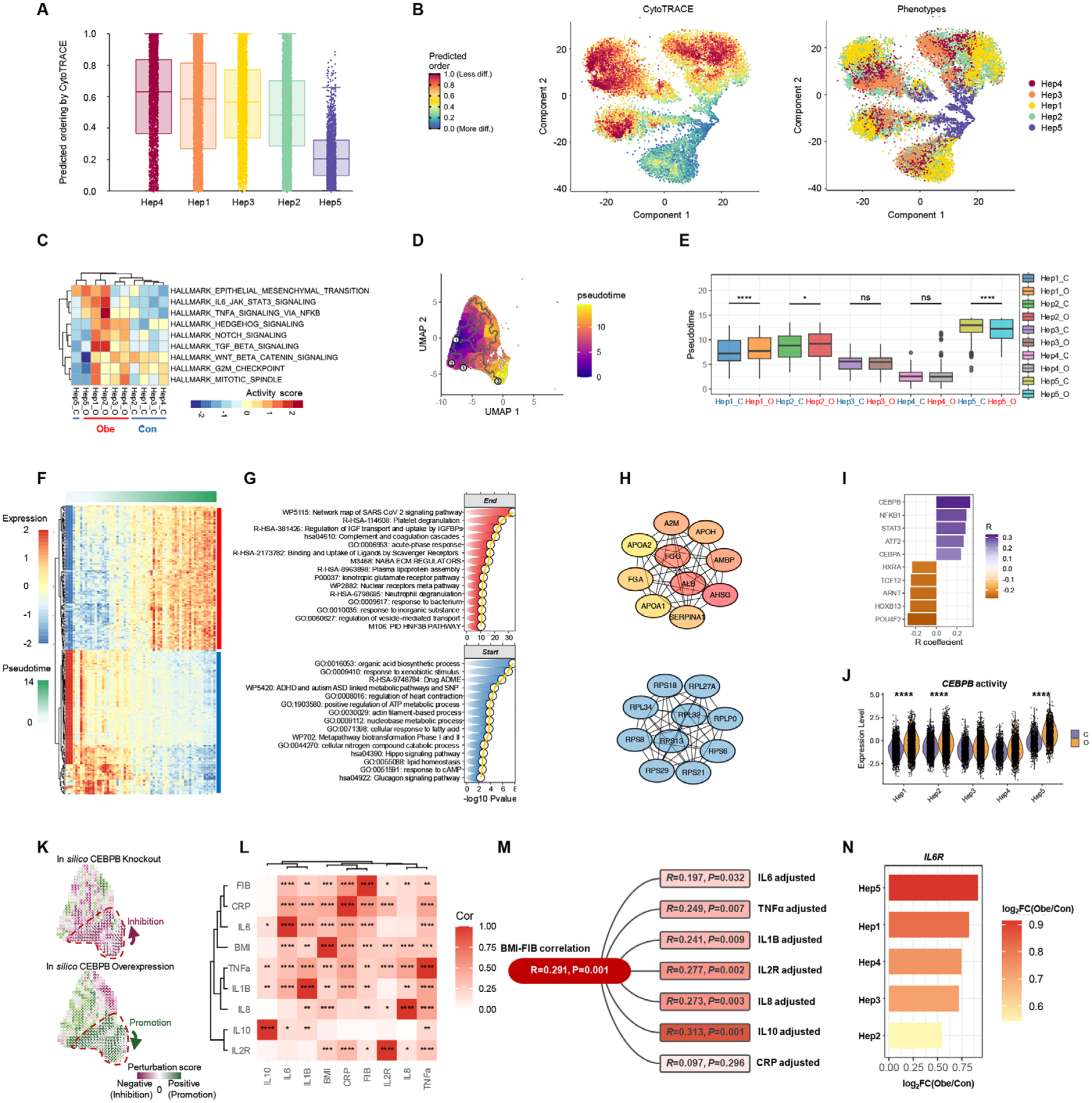
Enhanced differentiation from other hepatocyte clusters to Hep5 in obesity. (A) Predicted ordering by CytoTRACE of the five hepatocyte clusters, showing Hep5 having the lowest transcriptional diversity. (B) Plot of the CytoTRACE pseudo‐time order for the hepatocyte clusters. (C) Heatmap showing the pathway activity scores responsible for hepatocyte differentiation in the HALLMARK gene sets in hepatocyte clusters between obese (Obe) and control (Con) groups. (D) UMAP plot showing hepatocytes labeled by pseudotime value with trajectory from Monocle3. Pseudotime trajectories are visualized by color shading from purple to yellow for hepatocytes, as derived from Monocle3. (E) Bar plots of pseudotime values for all five hepatocyte clusters (Hep1‐5) in the control (C) and obese (O) groups. Between group comparisons of pseudotime values within the same cell cluster were performed using the Wilcoxon test. Statistical significance was determined at the following levels: *p < 0.05; ****p < 0.0001. (F) Heatmap showing the scaled expression of 100 genes positively and 100 genes negatively correlated with pseudotime. (G) Pathway enrichment results of 100 genes positively (upper panel) and 100 genes negatively (lower panel) correlated with pseudotime using the Metascape online tool. (H) PPI network of hub genes identified among the 100 genes positively (upper panel) and 100 genes negatively (lower panel) correlated with pseudotime, as determined by cytoHubba in Cytoscape software. (I) Transcription factors whose activities were most positively and most negatively correlated with pseudotime value in each cell, shown with Pearson *R* coefficients. (J) Violin plot showing CEBPB activity in each cell from Hep1‐5 in different groups (C, control group; O, obese group). Wilcoxon test, ****P < 0.0001. (K) *CEBPB* knockout and overexpression simulation vector field with perturbation scores inferred by the machine‐learning‐based approach, CellOracle. CellOracle derives perturbation scores by assessing the difference between the perturbation‐induced transition vector and the direction of cell differentiation. (L) Heatmap showing the Spearman correlations between BMI, plasma fibrinogen, C‐reactive protein, and inflammatory factor profiles (IL6, interleukin 6; IL1B, interleukin 1B; IL2R, interleukin 2 receptor; IL8, interleukin 8; IL10, interleukin 10; TNFα, tumor necrosis factor α) in 178 obese individuals. Significant correlations with adjusted *p*‐value < 0.05 are labeled in the heatmap with asterisks. *adjusted *p* < 0.05; **adjusted *p* < 0.01; ***adjusted *p* < 0.001; ****adjusted *p* < 0.0001. (M) Summary of partial Spearman correlation of BMI and plasma fibrinogen, adjusted by blood inflammatory factor profiles in obese individuals. (N) Differential expression of interleukin 6 receptor (IL6R) across the hepatocyte clusters between obese and control groups.

Although the proportion of Hep5 increased, its cell cycle distribution, stemness, and proliferation did not appear altered by conditions of obesity. We therefore assessed the differentiation potentials of each hepatocyte cluster based on transcriptional diversity in the CytoTRACE framework, in which differentiation is robustly related to decreased transcriptional diversity [26]. Among hepatocyte clusters, Hep5 exhibited the lowest transcriptional diversity (Figure 4A), suggesting a later differentiation state. Visualization of CytoTRACE results by *t*‐distributed stochastic neighbor embedding (*t*‐SNE) revealed that the Hep1–Hep4 clusters could all potentially transform into the Hep5 cluster (Figure 4B). Using the recently developed PhyloVelo software to infer cell differentiation trajectories and evolutionary lineages from scRNA‐seq data [27], we observed similar results that indicated other hepatocyte clusters had the capacity to differentiate into Hep5 (Figure S6A, Supporting Information). We also evaluated the activity of hepatocyte differentiation‐related pathways, including epithelial‐mesenchymal transition, IL6‐JAK‐STAT3 signaling, NFKB‐dependent TNFA signaling, hedgehog signaling,  Notch signaling, TGF‐beta signaling, WNT‐beta signaling, G2M checkpoint and mitotic spindle [18], in the Hallmark gene sets defined by the Molecular Signatures Database (MSigDB) across the five hepatocyte clusters, as well as between the obese and control groups. These pathway activity comparisons between the obese and control groups indicated that the activities of these pathways were markedly elevated in the obese group (Figure 4C), especially in Hep1‐4 clusters, which suggested that Hep1‐4 cells had enhanced capacity for differentiation in the obese group.

To further characterize the prevalence of Hep1‐4 differentiation into Hep5, we conducted pseudotime trajectory analysis using Monocle3 and selected the trajectory starting point based on our above CytoTRACE analysis. The predicted pseudotime trajectory from Hep1‐4 subpopulations into Hep5 cells (Figure 4D) was enhanced in obese individuals (Figure S6B, Supporting Information). We compared pseudotime values of each of the five hepatocyte clusters between the obese and control groups (Figure 4E). This analysis revealed that the pseudotime values of Hep1 and Hep2 were significantly higher in the obese group, suggesting these cells show a greater tendency to differentiate toward Hep5. Conversely, the pseudotime values of the Hep5 cluster were significantly lower in the obese group, suggesting that Hep5 cells in obese liver samples are predominantly derived from the differentiation of other hepatocyte clusters. Considering the limitation of Monocle3 to account for the possible presence of multiple concurrent gene processes in the same cell cluster, we also applied the relatively new GeneTrajectory [28] approach to infer the trajectories of individual genes and define the pseudotime order of gene expression patterns in hepatocytes of the obese and control groups. We grouped the genes into seven bins according to their pseudotime order. Plots of gene bins for both groups revealed that genes expressed at the terminal end of the trajectory were predominantly expressed in Hep5 cells (Figure S6C,D, Supporting Information).

To infer which signals could potentially trigger Hep5 differentiation, we deciphered the differentiation pathway activation and inhibition dynamics in pseudotime by identifying genes with the strongest correlation to pseudotime order (Figure 4F). Functional pathway enrichment analysis indicated that ‘platelet degranulation’ and ‘complement and coagulation cascades’ were activated, while the ‘hippo signaling pathway’ was inhibited over the pseudotime progression (Figure 4G). The STRING database was then used to construct protein–protein interaction (PPI) networks for the 100 genes with strongest positive and strongest negative correlations with pseudotime order, which revealed that FGA and FGG, along with some ribosomal proteins served as hub proteins in these networks (Figure 4H). Together, these results illustrated a hepatocyte cell fate trajectory in which the Hep5 subpopulation was increased by differentiation of Hep1‐4 clusters under conditions of obesity.

### 2.6 Enhanced IL6/CEBPB Signaling Induces Hep5 Differentiation in Obese Individuals

As the differentiation process is typically activated by a transcription factor (TF)‐driven signal cascade, we used DoRothEA software to infer gene regulatory networks, and especially TFs, involved in obesity‐associated hepatocyte differentiation. Mining the transcriptomic data for TFs with predicted activities most relevant to pseudotime progression identified the five most positively and most negatively correlated TFs (positive: *CEBPB*, *NFKB1*, *STAT3*, *ATF2*, and *CEBPA*; negative: *POU4F2*, *HOXB13*, *ARNT*, *TCF12*, and *RXRA*; Figure 4I). In particular, the regulatory activity of *CEBPB*, also known as *NFIL6* (nuclear factor IL6 regulated), shared the most significant positive correlation with pseudotime order (*R* = 0.33, *P *< 1 × 10^−4^, Figure 4I; Table S6, Supporting Information). When comparing the obese and control groups, *CEBPB* activity as a transcription factor was significantly upregulated in Hep1, Hep2, and Hep5 clusters of the obese group, most prominently in Hep5 (Figure 4J). Leveraging CellOracle, a computational framework that simulates transcriptional perturbations from single‐cell multi‐omics‐derived gene regulatory networks, we modeled *CEBPB* knockout and overexpression. By calculating a perturbation score that quantifies the alignment between simulated state transitions and the native differentiation trajectory, our simulations revealed that *CEBPB* knockout suppressed, whereas its overexpression promoted, cellular differentiation toward the Hep5 state (Figure 4K).

Noting that the TFs most closely associated with pseudotime progression were involved in classical inflammatory factor signaling pathways, we recruited an additional 178 obese individuals with blood inflammatory factor profile data, including IL6, IL1B, IL2R, IL8, IL10, and TNFα (Table S7, Supporting Information). Correlation analyses between these inflammatory factors and BMI, fibrinogen (FIB) and the classical acute‐phase response protein, C‐reactive protein (CRP), as well as between FIB and CRP, revealed that IL6 levels shared strong positive correlation with BMI (Figure 4L). To investigate which inflammatory factor played the largest role in BMI‐induced elevation of fibrinogen, we performed a partial Spearman correlation analysis between BMI and fibrinogen, adjusting for each inflammatory factor separately (Figure 4M). The results showed that the correlation between BMI and fibrinogen was significantly attenuated after adjusting for IL6, whereas adjusting for the other inflammatory factors had little effect on the correlation results (Figure 4M). This finding suggested that IL6 could potentially play a profound role in the BMI‐induced increase in fibrinogen. Binding of IL6 to IL6 receptor (IL6R) is a classical signaling for IL6 signal transduction. Therefore, to further explore which hepatocyte clusters exhibited the most pronounced response to IL6, we probed the expression levels of the IL6R and found that it was upregulated to the greatest extent in Hep5 cells (Figure 4N). Computational predictions using JASPAR identified CEBPB as a high‐confidence transcriptional regulator at the promoter region of IL6R (Figure S7A,B, Supporting Information). Supporting this, differential transcription factor activity analysis in Hep5 ranked CEBPB as the second most significantly upregulated transcription factor, suggesting that CEBPB was also the key factor for IL6R induction in Hep5 (Figure S7C, Supporting Information). Overall, these results suggested that an obesity‐induced elevation in IL6 levels could drive the differentiation of Hep1‐4 populations into Hep5 through activation of IL6‐CEBPB signaling.

### 2.7 CEBPB Inhibitor Helenalin Acetate Reverses the Aberrant Expression of Hepatocyte Differentiation‐Related Genes Induced by IL6

**FIGURE 5:**
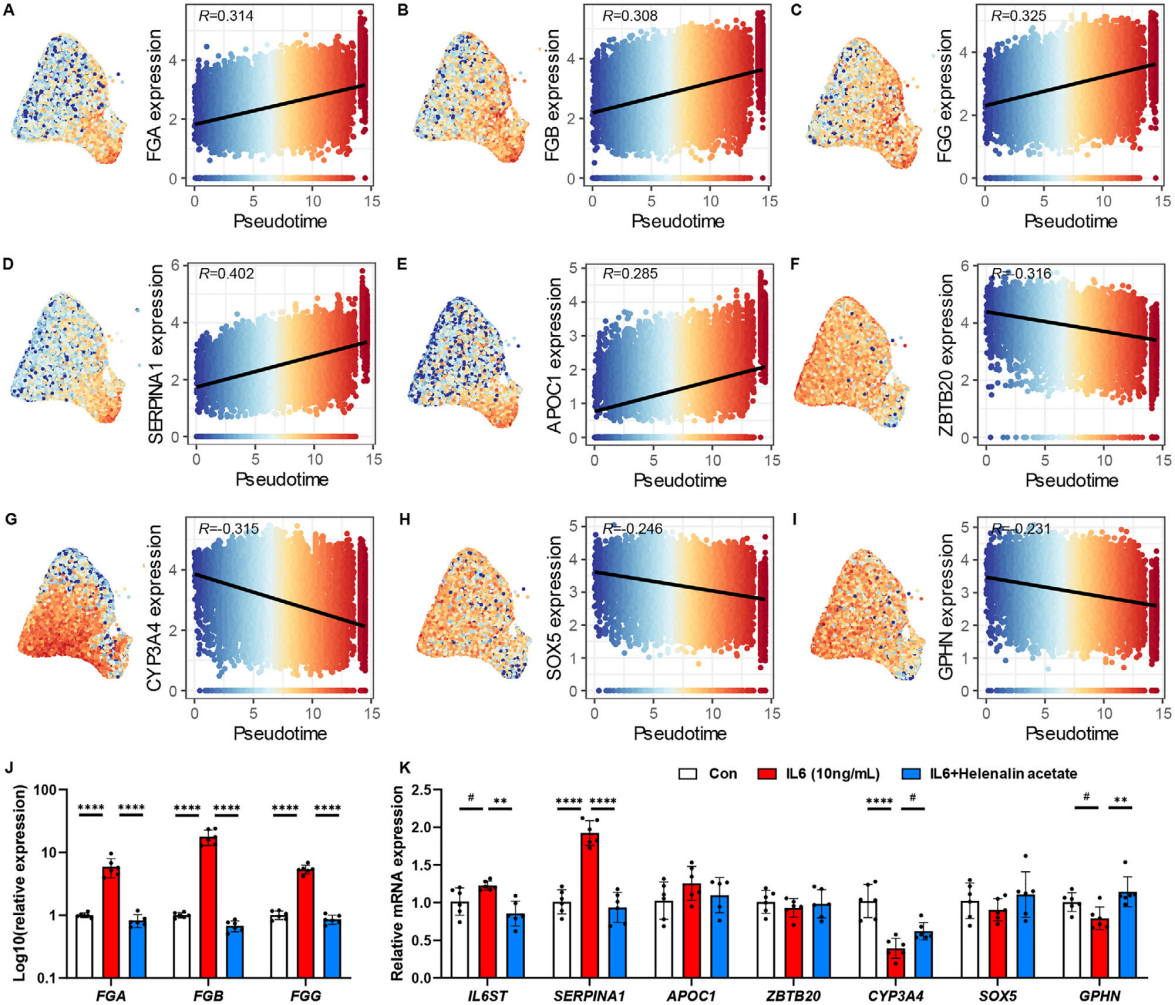
CEBPB inhibition by helenalin acetate reverses the aberrant expression of hepatocyte differentiation‐related genes induced by IL6. (A–I) The expression of key genes correlated with pseudotime on the UMAP coordinates of the hepatocytes. Colors represent different gene expression levels from red (high level) to blue (low level). Scatter plot showing the Pearson correlation of gene expression levels and pseudotime values in each hepatocyte. Correlation coefficients were labeled. (J,K) Fibrinogen genes (*FGA*, *FGB*, and *FGG*), IL6 downstream target gene (*IL6ST*), genes positively correlated with pseudotime (*SERPINA1*, *APOC1*, *FGA*, *FGB*, and *FGG*) and genes negatively correlated with pseudotime (*ZBTB20*, *CYP3A4*, *SOX5*, and *GPHN*) mRNA expression after IL6 (10 ng mL^−1^) exposure or combined IL6 (10 ng mL^−1^) and CEBPB inhibitor (Helenalin acetate, 1 nM) treatment in HepG2 cells over 48 h. Data are presented as mean ± S.E.M., with individual replicates shown (*n* = 5–6 per group). (One‐way ANOVA followed by Tukey's multiple comparisons test; *P < 0.05; **P < 0.01; ***P < 0.001; ****P < 0.0001). Trends are indicated by ^#^ *P* < 0.10. Normalized to *ACTB* expression.

Recent advancements in phase 2b trials investigating IL6 inhibition through monoclonal antibodies have shown great promise in managing inflammation‐related conditions and atherosclerosis, while also achieving notable reductions in serum fibrinogen levels [29, 30]. However, these IL6 monoclonal antibodies are unavailable and also unsuitable for in vitro experiments. Therefore, we hypothesize that targeting CEBPB may also hold potential for reducing fibrinogen levels. Through our search, we identified helenalin acetate (HA), a naturally occurring sesquiterpene lactone found in certain plants, which acts as a CEBPB inhibitor [31]. Therefore, to investigate the impact of the IL6‐CEBPB axis on hepatocyte differentiation, we conducted in vitro treatments on human hepatocyte cells using 10 ng mL^−1^ IL6 and IL6 combined with 1 nM HA. We then assessed the expression of genes closely associated with hepatocyte differentiation. IL6ST, a canonical signal transducer of IL6, was upregulated at the mRNA level in IL6 treatment and rescued in IL6 combined with HA treatment (Figure 5K). Genes such as *FGA*, *FGB*, *FGG*, *SERPINA1*, and *APOC1* (Figure 5A–E) showed significant positive correlations with pseudotime values, while *ZBTB20*, *CYP3A4*, *SOX5* and *GPHN* (Figure 5F–I) were significantly negatively correlated with pseudotime values. In vitro IL6 exposure, mRNA expressions of *FGA*, *FGB*, *FGG*, and *SERPINA1* (Figure 5J,K) were remarkably enhanced, and mRNA expressions of *ZBTB20*, *CYP3A4*, *SOX5*, and *GPHN* (Figure 5J,K) were suppressed. Following the addition of HA, we observed a rescue in the aberrant expression of hepatocyte differentiation‐related genes. To further validate the mechanistic role of *CEBPB* in obesity‐associated hypercoagulability, we performed siRNA‐mediated knockdown of *CEBPB* in HepG2 cells (Figure S8, Supporting Information). In addition, primary human hepatocytes were treated with physiologically relevant IL6 concentrations (10 ng mL^−1^) alone or in combination with HA or *CEBPB* siRNA; an elevated IL6 condition (50 ng mL^−1^) was also included for comparison, followed by transcriptomics analysis (Figures S9 and S10, Supporting Information). Genes upregulated by IL6 but subsequently suppressed by HA or siR‐*CEBPB* were predominantly enriched in pathways related to endoplasmic reticulum protein processing, complement and coagulation cascades, and protein export, mirroring Hep5's functional signature (Figures S9 and S10, Supporting Information).

Among these dynamically regulated genes, the top‐ranked candidates included fibrinogen‐related genes (*FGA*, *FGB*, and *FGG*), *CEBPB* and other pseudotime‐positively correlated genes, were activated by IL6 and subsequently inhibited by HA or siR‐*CEBPB*. These pseudotime‐negatively correlated genes were suppressed by IL6 and subsequently activated by HA or siR‐*CEBPB* (Figures S9 and S10, Supporting Information).

Overall, this study demonstrates that IL6 can induce corresponding expression changes in pseudotime progression‐related genes associated with hepatocyte differentiation, as identified in our snRNA‐seq data. These changes are attenuated upon inhibition of CEBPB, suggesting that the IL6‐CEBPB axis plays a crucial role in promoting hepatocyte differentiation in obesity. This finding also indicates that blocking this pathway may be important in reducing fibrinogen levels and lowering the risk of hypercoagulability‐related diseases in obese patients with elevated fibrinogen levels.

## 3 Discussion

We generated a bulk RNA‐seq and snRNA‐seq hepatocyte atlas from liver biopsies of healthy‐weight and obese individuals, which provided a transcriptomic and cellular landscape perspective of hepatocyte heterogeneity in obesity. Further, through Mendelian randomization, we established a causal relationship between elevated BMI and increased fibrinogen levels, resulting in an obesity‐related hypercoagulation phenotype.

As a growing global health issue, obesity leads to hypercoagulability, increasing risk of various life‐threatening conditions. However, these studies are largely based on clinical correlation analysis. Fibrinogen, encoded by the *FGA*, *FGB*, and *FGG* genes [32], has been identified as an independent risk factor in cardiovascular diseases (CVDs) [33, 34, 35], and its transcription, inducible by IL6 signaling [32, 36], is controlled by TFs such as hepatocyte nuclear factor (HNF)‐1 and CEBPs. Despite its observed upregulation in obesity [37, 38, 39], the underlying mechanisms responsible for increased fibrinogen secretion remain unclear. In our study, Mendelian randomization indicated that BMI shared a significant causal relationship with only fibrinogen in both the UK and GIANT databases. Facilitated by snRNA‐seq results from healthy and obese liver samples, we found that Hep5, the hepatocyte population responsible for secreting the highest levels of coagulation factors, was increased in both quantity and quality, supported by the significantly higher number and expression level of fibrinogen genes, and likely triggered by enhanced differentiation into Hep5 cells induced by activation of the IL6/CEBPB signaling pathways.

IL6 is a cytokine that participates in immune, metabolic, and liver regeneration functions under physiological conditions [40]. It is strongly associated with chronic inflammatory states, including low‐grade inflammation associated with obesity and type 2 diabetes mellitus [41]. Recent systematic multi‐omics studies found that high variance in serum IL6 could be largely explained by BMI, further highlighting the intimate link between IL6 and obesity. In addition, IL6 levels were closely associated with coagulation phenotype. In patients with COVID‐19, IL6 signaling drives coagulopathy and hepatic endotheliopathy, leading to liver injury [42, 43]. Here, we determined that the TFs most closely associated with the pseudotime progression were all involved in inflammatory response pathways. Among the six major inflammatory factors detected in the blood of our obesity cohort, IL6 showed the strongest significant positive correlation with BMI and plasma fibrinogen. CRP (C‐reactive protein), as a classic downstream product of the IL6 signaling pathway, is often used as a marker of IL6 signaling activity [44]. Given that both fibrinogen and CRP are products of the IL6 signaling pathway and are both secreted by hepatocytes, their strong correlation in the blood of our obesity cohort further supports the notion that the increased fibrinogen levels in obesity were driven by IL6 activation, which leads to enhanced secretion by hepatocytes. In Hep5 cells, IL6R expression was mostly upregulated in the obese individuals compared with other hepatocyte types. Additionally, genome‐wide association analysis (GWAS) identified IL6R as the closest gene to several genetic loci associated with circulating fibrinogen. These findings suggested that IL6 plays a critical role in obesity‐related hyperfibrinogenesis.

Recent advances in IL6 inhibition using monoclonal antibodies have shown potential for managing inflammation‐related conditions and atherosclerosis. In a double‐blind, randomized, placebo‐controlled, phase 2 trial in patients at high atherosclerotic risk, IL6 inhibition with the monoclonal antibody ziltivekimab, which directly targets the IL6 ligand, led to markedly reduced expression of atherosclerosis‐related biomarkers of inflammation and thrombosis [29]. Additionally, ziltivekimab administration led to a dose‐dependent reduction in fibrinogen levels. Furthermore, another monoclonal antibody targeting the IL6 ligand, clazakizumab, was also shown to reduce serum fibrinogen levels in a phase 2b dose optimization study. For IL6 antibodies, analysis of phase II trials indicates that both ziltivekimab and clazakizumab demonstrated therapeutic efficacy in patients with chronic kidney disease (CKD) or maintenance dialysis recipients exhibiting cardiovascular comorbidities (cardiovascular disease/diabetes) accompanied by elevated high‐sensitivity C‐reactive protein (hs‐CRP; >2 mg/L) [29, 30]. Both agents show significant anti‐inflammatory effects with dose‐dependent reductions in hs‐CRP and fibrinogen levels. These antibodies also lead to decreases in serum amyloid A (SAA), secretory phospholipase A2 (sPLA2), and lipoprotein(a) concentrations. Notably, clazakizumab treatment was associated with increased serum albumin levels, potentially reflecting its dual roles in anti‐inflammatory and nutritional modulation. Regarding safety, these antibodies exhibit favorable profiles without sustained severe hematologic toxicities or thrombocytopenia, though they show differential metabolic effects—clazakizumab causes modest cholesterol elevation while ziltivekimab shows a neutral effect on lipid metabolism. Conversely, ziltivekimab demonstrated minimal impact on platelet counts and hepatic enzymes. These findings collectively highlight the potential of monoclonal antibodies against IL6 to modulate fibrinogen synthesis [30], and support the use of these drugs to inhibit fibrinogen synthesis and mitigate excessive hepatocyte differentiation into the Hep5 subtype in obese patients. This therapeutic strategy could address obesity‐related hypercoagulation by targeting underlying inflammatory pathways and reducing fibrinogen levels.

Regarding CEBPB inhibitors, helenalin acetate is a natural sesquiterpene lactone with anti‐inflammatory and anti‐cancer properties [31]. Current research primarily explores its applications in cancer and infectious diseases, revealing broad therapeutic potential [45]. In infectious models, helenalin acetate induces mitochondrial dysfunction, characterized by reduced membrane potential and structural damage, as confirmed by transmission electron microscopy, which revealed marked organelle swelling [46]. Notably, it does not affect plasma membrane permeability or reactive oxygen species (ROS) levels but enhances nitric oxide production in peritoneal macrophages, potentially boosting anti‐parasitic activity against intracellular amastigotes. These findings suggest its promise as an anti‐leishmanial scaffold for further optimization. In cancer studies, helenalin acetate exhibits wide‐ranging effects, including the upregulation of differentiation‐associated genes, induction of cell death, and suppression of self‐renewal in acute myeloid leukemia (AML) cells [47, 48]. It also inhibits proliferation and migration in lung adenocarcinoma [49]. Moreover, it has the capacity for metabolism regulation and attenuates adipocyte differentiation in preadipocytes [31, 50, 51]. Despite its multi‐tissue efficacy, its proliferation‐inhibiting cytotoxicity calls for careful dose selection. Given this compound's natural origin, the development of targeted delivery systems, particularly to the liver, could enhance its applicability in obesity‐related contexts by selectively inhibiting aberrant hepatocyte differentiation.

The liver is a vital organ with diverse functions, including detoxification, secretion of plasma proteins, and metabolism, primarily mediated by hepatocytes [52, 53]. A tightly regulated transcriptomic program is required for the formation of functional hepatocytes [53]. Mature hepatocytes derived from hepatic progenitor‐like cells have also been observed in vivo under pathological conditions such as acute injury, MASLD, hepatitis, and cirrhosis [17, 54]. A suite of TFs and signaling pathways, including the HNF family, CEBP family, Wnt/β‐Catenin signaling, Notch signaling, Hedgehog signaling, and others [17, 54, 55], regulate cell fate determination and differentiation into mature hepatocytes. IL6 has been identified as a key modulator of hepatocyte differentiation and regeneration [18, 40]. In our study, these differentiation‐related pathways were highly activated in hepatocytes of obese individuals. Considering that cell cycle, proliferation, and stemness markers were expressed at comparable levels between Hep5 and other hepatocyte clusters, it is likely that the increased abundance of Hep5 cells was due to differentiation from other hepatocyte types. This speculation was confirmed by pseudotime trajectories inferred from cell‐typing and gene expression data. Among all TFs detected in RNA‐seq data, CEBPB activity showed the highest correlation with pseudotime values, and both its activity and transcription levels were significantly elevated in the obese group, suggesting a potentially instrumental role in driving Hep5 differentiation in obesity. CEBPB reportedly participates in liver regeneration and terminal hepatocyte differentiation [56, 57, 58]. Moreover, CEBPB, which is also known as nuclear factor IL6 regulated (NF‐IL6), is a classical downstream target of IL6 signaling and can enhance IL6 production [59, 60]. These results further support the central role of IL6 in obesity.

Our snRNA‐seq analyses revealed that Hep5 exhibited marked upregulation of IL6R, a finding that may reflect its specialized role in obesity‐associated fibrinogen hypersecretion. Through promoter analysis and transcriptional regulatory network inference, we identified CEBPB as a putative driver of IL6R expression in Hep5. Intriguingly, we propose a feedforward mechanism whereby obesity‐induced IL6 could activate CEBPB, thereby amplifying IL6R expression in Hep5. This positive feedback loop might hypersensitize Hep5 to microenvironmental IL6, potentiating metabolic reprogramming and fibrinogen production. These findings align with emerging evidence of metabolic‐inflammatory crosstalk in hypercoagulation pathogenesis and highlight Hep5 as a potential node for therapeutic intervention.

Despite these findings, several limitations should be acknowledged. First, the initial snRNA‐seq cohort size remains limited. It is important to note that our findings are derived from a cohort of premenopausal females, which was essential to control for confounding metabolic factors. Consequently, the results may not be directly generalizable to males or postmenopausal females, highlighting the need for future studies in more diverse cohorts. Second, although our transcriptional data strongly suggest an IL6/CEBPB regulatory axis, direct experimental validation using lineage‐tracing approaches and IL6 knockout models would further strengthen the mechanistic evidence.

In summary, Mendelian randomization analysis and the comprehensive transcriptomic atlas of obese livers together uncovered the causal relationship between BMI and elevated fibrinogen levels, as well as the increased likelihood of differentiation into fibrinogen‐secreting hepatocytes, which may be alleviated with therapeutic antibodies targeting IL6. This study can serve as a reference to guide further research and development of advanced therapeutic strategies for treating hypercoagulation and other obesity‐related disorders.

## 4 Materials and Methods

### 4.1 Sample Collection and Ethical Statement

The liver tissues were isolated from patients undergoing surgery for benign liver tumors and sleeve gastrectomy for morbid obesity from 2021 to 2022. Individuals with a history of diabetes mellitus, severe hepatic dysfunction, and/or prior use of metabolic medications were excluded from the study. All participants were age‐matched and female (premenopausal, non‐pregnant) to minimize sex‐based metabolic variability. The clinical characteristics and comorbidities were summarized in Table S8, Supporting Information. After isolation, liver tissue samples were immediately subjected to snap‐frozen in liquid nitrogen, followed by bulk RNA‐seq and snRNA‐seq. Our research received approval from the Ethics Committee of Qilu Hospital of Shandong University (KYLL‐2017‐073), and all patients were informed and provided consent.

Liver histopathology was evaluated using standardized scoring systems: Fibrosis was classified according to a 5‐tier grading system (F0–F4), while steatosis (0–3), lobular inflammation (0–3), and hepatocyte ballooning (0–2) were scored in compliance with the NASH Clinical Research Network (CRN) criteria. These components were integrated to calculate the NAFLD Activity Score (NAS), which ranges from 0 to 8 points. Obese patients were stratified by MASLD severity: mild (NAS < 5) vs. severe (NAS ≥ 5). Dyslipidemia is defined as the presence of at least one of the following: hypertriglyceridemia, hypercholesterolemia, hyper‐LDL‐cholesterolemia, or hypo‐HDL‐cholesterolemia. Hypertriglyceridemia is defined as triglycerides more than 1.7 mmol/L. Hypercholesterolemia is defined as cholesterol more than 5.2 mmol/L. Hyper‐LDL‐cholesterolemia is defined as LDL more than 3.35 mmol/L. Hypo‐HDL‐cholesterolemia is defined as HDL less than 1 mmol/L. Obese patients stratified by lipid profiles: dyslipidemia (abnormal lipid metabolism) versus normal (without dyslipidemia).

Information on BMI, fibrinogen, C‐reactive protein, and inflammatory factor profiles in blood was collected from a cohort of 178 obese individuals undergoing sleeve gastrectomy for morbid obesity (Table S7, Supporting Information).

### 4.2 Instrumental Variable Selection and Mendelian Randomization Analysis

Single nucleotide polymorphisms (SNPs) associated with the BMI trait were meticulously selected as instrumental variables (IVs) from the IEU Open GWAS project (https://gwas.mrcieu.ac.uk/) and the GWAS Catalog (https://www.ebi.ac.uk/gwas/). Data on common coagulation outcomes were acquired by selecting common coagulation‐related parameters, including 13 types of coagulation factors and mean platelet volume, platelet count, platelet distribution width, and d‐dimer measurement. Table S1, Supporting Information, summarizes the sources of exposure and outcome data. To address potential confounders, we employed LDlink (https://ldlink.nih.gov/?tab = home) to scrutinize the instrumental variables for any associations with known confounding factors (e.g., smoking, alcohol consumption, type 2 diabetes, inflammatory markers, liver and kidney disease) (Table S1, Supporting Information). This crucial step helped us eliminate variables that might introduce bias into our analysis, ensuring that our instrumental variables were not confounded with outcomes unrelated to the exposure of interest. To assess the potential for weak instrumental variable bias, we calculated the *F*‐statistics for each IV. The *F*‐statistics were greater than 10, indicating no evidence of weak instrumental bias (Table S1, Supporting Information). An *F*‐statistics below 10 indicates a weak instrument, which was excluded from the analysis. We applied MR Pleiotropy Residual Sum and Outlier (MR‐PRESSO) to monitor the potential horizontal pleiotropy effect. For each SNP, the MR‐PRESSO outlier test calculated a *p*‐value for its pleiotropy significance, whereas the MR‐PRESSO global test calculated a *p*‐value for overall horizontal pleiotropy. When the *p*‐value of MR‐PRESSO global test is greater than 0.05, it indicates the absence of horizontal pleiotropy. The list of SNPs remaining after removing pleiotropic SNPs was used for the subsequent MR analysis. The primary analysis employed the inverse‐variance weighted (IVW) MR method, while sensitivity analyses were conducted using MR‐Egger, weighted median, simple mode, and weighted mode (Table S1, Supporting Information).

Cochran's *Q* statistics were employed to evaluate heterogeneity. If the *p*‐values exceeded 0.05, indicating no heterogeneity, the fixed‐effects IVW approach was adopted as the primary method. Conversely, if significant heterogeneity was detected (*p* < 0.05), the random‐effects IVW approach was utilized.

We conducted two‐sample MR analyses to investigate the potential causal relationships between the BMI trait and those coagulation outcomes using the TwoSampleMR package in R software [61]. The IVW method was predominantly used for MR analysis, providing a consistent estimate of the association between exposure and risk of outcomes when the IVs were not pleiotropic [62]. Association estimates were presented as beta with corresponding 95% confidence intervals (CI).

### 4.3 Single Nucleus Extraction

The frozen tissues from two controls and two obese individuals were supplemented with nucleus separation mix and homogenized on ice. After filtration 40 µm cell strainer, the mixture was centrifuged at 200 ×*g*, 4°C for 2 min. The supernatant was then centrifuged at 500 ×*g* at 4°C for 5 min, and the nuclei were present in the sediment and resuspended with PBS mix. After being labeled with DAPI, the nuclei were evaluated and counted under a fluorescence microscope.

### 4.4 Single Nucleus RNA‐seq

After the nuclei suspension preparation, the nuclei were loaded immediately into the microchip with Singleron Matrix. After the purified cDNA passed quality control, the snRNA‐seq libraries were generated from cDNA according to the GEXSCOPE Single Nucleus RNA Library Kit Handbook (SD Microchip‐Automated‐2 RXNs/16 RXNs, V2) in Singleron Biotechnologies, Nanjing, China. Fragmentation program and adaptor ligation were undertaken as per the manufacturer's protocol. AMPure XP (Illumina) was used for purification. The library quality control was done by using the QuBit 1× dsDNA HS assay kit. GEXSCOPE Single Cell RNA Library Kit produced standard Illumina paired‐end constructs beginning with P5 and ending with P7. 60 bp Barcodes (27 bp unique sequence plus spacers) were located right at the beginning of Read1, while an 8 bp i7 sample index sequence was positioned at the beginning of Read 2. P5 and P7 were standard Illumina sequencing primer sites used in paired‐end sequencing. Read 1 was used to resolve 60 bp Barcodes and 12 bp UMIs, while Read 2 was used to obtain gene‐specific sequences. A standard Illumina FASTQ file was produced by sequencing these libraries. After quantification and normalization, the GEXSCOPE Single Cell RNA Library was denatured and diluted as recommended for Illumina sequencing platforms, and then sequenced on Illumina sequencers (Illumina).

### 4.5 Processing snRNA‐seq Data

Raw reads from snRNA‐seq were manipulated using the CeleScope (https://github.com/singleron‐RD/CeleScope) v1.9.0 pipeline to generate gene expression matrices. In brief, raw reads were first processed with CeleScope, low‐quality reads were removed with Cutadapt v1.17, and multiple A‐tails and adaptor sequences were trimmed. After cell barcode and UMI extraction, the reads were mapped onto the reference genome GRCh38 (Ensembl version 92 gene annotation) via STAR v2.6.1a [63]. Subsequently, we obtained UMI counts and gene counts of each cell with featureCounts v2.0.1 software [64], and generated expression matrix files.

### 4.6 Quality Control and Clustering of snRNA‐seq

We constructed Seurat objects from each data set using the Seurat v4.3.0 R package [65] and carried out quality control of the snRNA‐seq expression matrix. Nuclei with UMI counts of more than 20,000, gene counts of less than 200 or more than 5,000, and mitochondrial content of more than 20% were filtered out. Next, we normalized data using *SCTransform* function and performed integration with the vars.to.regress = ‘percent_mito’ parameter. We used the top 20 principal components with a resolution of 0.3 for clustering. The uniform manifold approximation and projection (UMAP) was applied for dimensional reduction and visualizing the datasets. Clustering was performed using the Seurat package with the Louvain algorithm at multiple resolution parameters (ranging from 0.1 to 1.0). To determine the optimal resolution, we employed the clustree v 0.5.1 package to visualize the relationships between clusters across consecutive resolutions [66]. The clustree diagram revealed stable clustering patterns between resolution 0.2 and 0.4, where minimal cross‐branching was observed, indicating robust partition boundaries. Based on biological plausibility and comparison with established liver single‐nucleus RNA sequencing studies (which typically identify 10–15 major cell populations), we selected a resolution of 0.3. This parameter provided an optimal balance between over‐clustering and under‐clustering, yielding 17 distinct cell populations (0‐16) that corresponded well with known liver cell types while maintaining clear separation between clusters in the UMAP space. Cluster 15 was annotated as erythrocyte‐derived due to near‐ubiquitous expression of hemoglobin genes (*HBA1* and *HBB*). Given that mature erythrocytes lack nuclei, their detection in single‐nucleus RNA‐seq data likely stems from residual cytoplasmic RNA in contaminating fragments. Therefore, cluster 15, marked by red blood cells, was removed for further analysis.

For sub‐clustering of Hep5 clusters, a Seurat object was constructed containing only the cells originating from this cluster, and the same procedures were applied with a resolution of 0.2.

### 4.7 Marker Genes Identification and Cell‐Type Annotation

Marker genes of each cluster were identified by using *FindAllMarkers* function. Parameters for this function were set by default as follows: genes detected in at least 25% of cells and a differential expression threshold of absolute log_2_ fold‐change > 0.25. Significant genes were determined with *p*‐value < 0.05 following Bonferroni correction. The annotation of the cell type was applied based on the top marker genes of each cluster manually. Top marker genes (ranked by fold change and adjusted *p*‐value) were cross‐referenced with canonical cell‐type signatures from liver single‐cell/nucleus studies (e.g., LSEC: *LYVE1*, *STAB2*; hepatocytes: *CYP2B7P*, *LBP*; Kupffer cells: *VSIG4*, *FCGR3A*; Endo: *VWF*, *PECAM1*). We queried the CellMarker 2.0 database (http://bio‐bigdata.hrbmu.edu.cn/CellMarker/) to resolve ambiguous annotations, prioritizing markers with tissue‐specific support. Considering potential biases arising from data analysis, we aimed to further validate annotations and performed pathway analysis on cluster‐specific markers using the Metascape online tool for functional pathway enrichment. *FeaturePlot*, *DimPlot*, *DotPlot*, and *VlnPlot* functions were employed to visualize and iteratively validate marker genes.

Additionally, to account for potential inter‐individual variability among clinical samples, we performed systematic visualization of sample origins for each cell type to rule out the possibility that any cluster might be driven by outliers from specific donors. Our analysis confirmed balanced representation across samples, with no evidence of donor‐specific clustering patterns. Furthermore, we verified that batch effects were effectively corrected through SCT integration, ensuring our cell type annotations reflect true biological variation rather than technical artifacts or individual outliers.

### 4.8 Differential Expression Analysis

Differential expression (DE) analysis was performed using the Libra v1.0.0 R package [67], using *run*_de function using method Wilcoxon rank‐sum test. Genes with absolute log_2_ fold‐change > 1 and adjusted *p*‐value < 0.05 are considered differentially expressed genes (DEGs). The threshold for identifying DEGs was commonly used in omics studies, effectively controlling the false discovery rate to < 5% and maintaining adequate statistical power. A volcano plot was generated by the ggplot2 R package for DE visualization.

### 4.9 Correlation Analysis

The correlation analysis between BMI and other clinical indices or hepatocyte proportions was conducted using *corr.test* function in the psych R package [68], and the correlation coefficients and the *p*‐values adjusted by the Benjamini–Hochberg (BH) method were used for the correlation heatmap generation. Partial correlation was performed using *pcor.test* function from ppcor R package.

### 4.10 Pathway Analysis and Gene Set Scores

Pathway enrichment was undertaken using the Metascape [69] (https://metascape.org/gp/index.html#/main/step1) website using top marker genes of each cluster or using differential expression genes (DEGs). We used the irGSEA v 3.2.4 R package to calculate the activity of a custom gene set of interest, like a secreted protein gene set, stemness, or proliferative gene set. GSVA v1.48.3 R package [70] was utilized to calculate the activity scores of pathways in the Hallmark gene set.

### 4.11 Cell Cycle Analysis

*CellCycleScoring* function in the Seurat package was used to score cell cycle phases. Features associated with S phase and G2M phase were extracted from *cc.genes*.

### 4.12 CytoTRACE and Pseudotime Analysis

Single‐cell predictions of differentiation status from snRNA‐seq data were generated using *CytoTRACE* in CytoTRACE v 0.3.3 R package [26]. Single‐cell trajectory and pseudotime analyses were conducted using PhyloVelo software [27] according to the tutorial in https://github.com/kunwang34/PhyloVelo/blob/master/docs/source/notebook/getting_start.ipynb, and Monocle3 v1.3.4 R package [71] with the default parameters based on cells in Hep1‐Hep5 clusters. Pearson correlation analysis was performed between the pseudotime values inferred for each cell by Monocle3 and the activity scores of each TF or gene expression in these cells by *corr.test* function in the psych R package.

### 4.13 Prediction of Transcription Factor (TF) Activities

DoRothEA [72] is a gene regulatory network (GRN) containing transcription factor (TF)‐ target gene interactions. We performed DoRothEA using *run_viper* function in dorothea R package.

### 4.14 Gene Trajectory Inference Analysis

GeneTrajectory was a newly‐developed method for deconvolution of complex biological processes by identifying gene trajectories rather than cell trajectories [28]. GeneTrajectory analysis was performed according to the tutorial in https://github.com/KlugerLab/GeneTrajectory. In using *FindVariableFeatures* function, nfeatures was set at 1000. In using *GetGraphDistance* function, *K* value was set at 50. Coarse‐grain graph was performed by grouping cells into *N* = 1000 meta‐cells.

### 4.15 Construction of Protein–Protein Interactions Networks

To obtain a more elaborate and intuitive understanding of the protein interactions involving the most correlated genes with pseudotime, the online tool, Search Tool for Retrieval of Interacting Genes (STRING, https://www.string‐db.org/) was used to visualize the top 100 most positively or negatively correlated genes and to construct a protein‐protein interaction network. The hub genes of the PPI network were identified using the cytoHubba plug‐in in Cytoscape software (version 3.10.2), using the top 10 nodes ranked by MCC, and only the shortest paths were displayed.

### 4.16 Bulk RNA‐seq Analysis

Total RNA extraction was performed using frozen liver samples, ensuring the preservation of RNA integrity and minimizing contamination. Extracted RNA was converted into a complementary DNA (cDNA) library through reverse transcription, followed by fragmentation and adapter ligation to facilitate sequencing. Subsequently, prepared libraries were sequenced using Illumina Novaseq 6000 platform in Shanghai Applied Protein Technology Co., Ltd.

The raw image data files obtained from high‐throughput sequencing were transformed into raw sequencing sequences (sequenced reads) through CASAVA base calling analysis. We referred to these as Raw Data or Raw Reads, which were stored in FASTQ (abbreviated as fq) file format. We also performed analyses such as sequencing error rate distribution check, A/T/G/C content distribution check, and raw data composition to assess the quality of sequencing data. HISAT2 software [73] using the BWT algorithm was used for mapping clean reads onto the reference genome (http://ftp.ensembl.org/pub/release‐104/fasta/homo_sapiens/dna/Homo_sapiens.GRCh38.dna_sm.primary_assembly.fa.gz). The FPKM values for expression of each gene in each sample were calculated using the featureCounts software. The count values were normalized for the batch of samples for library construction and sequenced on a sequencer using the sva v3.48.0 R package [74] for removing the batch effect.

### 4.17 Cell‐Type Deconvolution Analysis

Cell‐type deconvolution analysis was conducted using CIBERSORT v0.1.0, BisqueRNA v1.0.5, and GSVA v1.48.3 R packages [70, 75, 76]. For CIBERSORT analysis, the matrix of heterogeneous mixed expression was generated according to the top 100 marker genes of each cell type cluster, and the number of permutations was set to 1000. For BisqueRNA analysis, we used the default parameters.

### 4.18 CellOracle Analysis

We employed CellOracle for GRN inference and in silico perturbation modeling. The analysis was conducted in accordance with the tool's standard protocol, guided by the code and documentation available at https://github.com/morris‐lab/CellOracle.

### 4.19 Spatial Transcriptomics Analysis

Spatial transcriptomics data of human normal liver were acquired under accession: GSE185477 and GSE243981 [14], and the data was loaded into Seurat using the *Load10X_Spatial* function. The data normalization was performed using *SCTransform* function. *FindNeighbors* function was applied using ‘pca’ reduction method and the top 30 principal components. *FindClusters* and *clustree* were both used for clustering of spatial transcriptomics data, and resolution was decided at 1.3.

According to the marker genes of different regions of hepatocytes and the histological image of spatial transcriptomics data [11, 14, 25], we finally defined these clusters into three clusters: central venous region (CV), interzonal region (IZ), and periportal region (PP). The *AddModuleScore* function was used for the gene set score calculation based on the published CV markers, IZ markers, and PP markers as well as the top 50 markers of each hepatocyte cluster (Hep1‐Hep5). *DimPlot*, *SpatialDimPlot*, and *SpatialFeaturePlot* functions were used for visualization.

For GSE243981 data analysis, we used human healthy liver spatial data via https://macparlandlab.shinyapps.io/healthylivermapspatialgui/, and visualized PP markers (*HAL*, *SDS*), CV markers (*GLUL*, *SLC1A2*), and fibrinogen genes.

### 4.20 Hepatocyte Clustering in External Validation

The liver single‐cell RNA‐seq datasets from GSE185477 and GSE180665 were downloaded, and hepatocytes derived from normal human liver tissues were extracted. Data integration, clustering, and marker gene identification were performed following the same procedures as described earlier in the Methods.

### 4.21 IL6R Promoter and Transcription Factor Prediction

IL6R promoter region (2 kb upstream) was analyzed using the UCSC Genome Browser (hg38). Transcription factor binding sites (TFBS) were predicted using the JASPAR CORE vertebrate database (2022 release) with a minimum score threshold of 450. High‐confidence CEBPB binding sites were validated through position weight matrix matching using the JASPAR database (https://jaspar.elixir.no/).

### 4.22 Immunofluorescence Staining

Human liver tissue sections were fixed in 4% paraformaldehyde (PFA) for 15 min, permeabilized with 0.1% Triton X‐100 (15 min), and blocked with 5% BSA (1 h, RT). Primary antibodies were incubated overnight at 4°C in a humidified chamber:

Anti‐APOE (Proteintech, cat# 66830‐1, 1:800)

Anti‐FGG (Proteintech, cat# 15841‐1, 1:100)

Anti‐GLUL (Marker of CV hepatocytes, Proteintech, cat# 66323‐1, 1:1000)

After PBS washes, sections were incubated with species‐matched secondary antibodies conjugated to Alexa Fluor 488/555/547 (Invitrogen, 1:500, 1 h, RT). Nuclei were counterstained with DAPI (5 min), and slides were mounted with ProLong Gold Antifade (Invitrogen).

Images were acquired using a confocal microscope (Zeiss LSM 900) under 20×/63× objectives. Whole‐slide scanning was conducted using 3DHISTECH digital pathology scanner.

### 4.23 Human Hepatocyte Culture and Treatment

HepG2 cells were cultured in a 37°C incubator with 5% CO_2_. The culture medium used was Dulbecco's Modified Eagle Medium (DMEM) supplemented with 10% fetal bovine serum and 1% Penicillin and Streptomycin. Experiments using cryopreserved primary human hepatocytes (PHHs) were performed in IPHASE Biosciences (China). For 48 h treatment, recombinant IL6 protein (MCE, HY‐P7044) was used at a concentration of 10 and 50 ng mL^−1^, and helenalin acetate (HA, MCE, HY‐114519) was used at 1 nM. siRNA transfection was performed using Lipofectamine 3000 (L3000, Invitrogen) according to the manufacturer's protocol. *CEBPB* siRNA was designed and synthesized by Tsingke Biological Technology, with the target sequence: siCEBPB‐1 sense, CCUCGCAGGUCAAGAGCAA, antisense, UUGCUCUUGACCUGCGAGG; siCEBPB‐2 sense CUGCCUUUAAAUCCAUGGA, antisense, UCCAUGGAUUUAAAGGCAG; siCEBPB‐3 sense, CCCGUGGUGUUAUUUAAAGAA, antisense UUCUUUAAAUAACACCACGGG. Total RNA was extracted and purified using an RNA‐quick extraction kit (ES‐RN001) according to the manufacturer's instructions. Following extraction, RNA was reverse transcribed using PrimeScript RT Reagent Kit with gDNA Eraser (Perfect Real Time, Takara), and quantitative PCR was performed using TB Green (Takara) to assess mRNA expression.

### 4.24 Quantifications and Statistical Analyses

Data were expressed as mean ± standard error of the mean (SEM), as indicated in the figure legends with n and statistical tests. Statistical analyses were performed using appropriate statistical tests as indicated in the figure legends.

To address potential metabolic confounders, we employed ANOVA to compare cell population proportions between obese and control groups while adjusting for these metabolic parameters, with particular focus on the obesity‐specific Hep5 subpopulation. All statistical analyses were performed using R version 4.3.1, with adjusted *P* < 0.05 considered statistically significant.

## Author Contributions

**Shumin Li**: methodology, investigation, visualization, funding acquisition, project administration, writing – original draft; writing – review and editing. **Gengchen Feng**: methodology, investigation, visualization, writing – original draft, writing – review and editing. **Xin Huang**: methodology, investigation, writing – original draft, writing – review and editing. **Xiao Teng**: visualization. **Ziyi Yang**: investigation. **Yue Liu**: investigation. **Yonghui Jiang**: visualization. **Tao Zhu**: methodology. **Teng Liu**: visualization. **Yunfei Xu**: methodology. **Chang Pan**: conceptualization, visualization, writing – review and editing. **Han Zhao**: conceptualization, funding acquisition, project administration, supervision, writing – review and editing. **Shaozhuang Liu**: conceptualization, supervision, writing – review and editing. **Shigang Zhao**: conceptualization, funding acquisition, supervision, writing – review and editing. **Zi‐Jiang Chen**: funding acquisition, project administration, supervision.

## Funding

This work was supported by the National Key Research and Development Program of China (2024YFC2707300, 2024YFC3405604), the National Natural Science Foundation of China (82421004, 32370916, 32588201, 82192874 and 32400713), the Shandong Provincial Key Research and Development Program (2024CXPT087), the Ningxia Hui Autonomous Region Key Research and Developmental Program (2024BEG02019), the Natural Science Foundation of Shandong Province for Excellent Youth Scholars (ZR2023YQ061), the CAMS Innovation Fund for Medical Sciences (2021‐I2M‐5‐001), the Program for Chang Jiang Scholars (Q2022144), the Taishan Scholars Program of Shandong Province (ts20190988), and the Fundamental Research Funds of Shandong University (2023QNTD004).

## Conflicts of Interest

The authors declare no conflicts of interest.

## Ethics Statement

Our research received approval from the Ethics Committee of Qilu Hospital of Shandong University (KYLL‐2017‐073), and all patients were informed and provided consent.

## Supporting information




**Supporting File**: exp270210‐sup‐0001‐SuppMat.zip.

## Data Availability

Data are available in the supplementary materials or upon reasonable request.
