# Supplementary material for: Hepatic Mechanisms and Therapeutic Target Underpinning Hypercoagulation in Obesity: Insights From Genetic and Transcriptomic Analyses
Source: Exploration (Beijing). 2026 Aug 13;6(4):70210. doi: 10.1002/exp2.70210 (PMC13473198; doi:10.1002/exp2.70210)
Supplement: Supplementary file 1 [file EXP2-6-70210-s001.zip › exp270210-sup-0009-Figures.docx]

Supplementary Materials for

**Hepatic Mechanisms and Therapeutic Target Underpinning Hypercoagulation in Obesity: Insights from Genetic and Transcriptomic Analyses**

**The supplementary materials file include:**

Supplementary Figures (Figs. S1 to S10)

Supplementary Tables (Table S1 to S8)

Supplementary Figures

Figure S1. Single nucleus RNA-seq (snRNA-seq) landscape of control and obese individual livers.

(**A**). Comparison of clinical characteristics between control (Con) and obese (Obe) individuals in Cohort 1. Data are presented as box plots showing the median and interquartile range (IQR), with individual data points shown. Statistical significance was determined using Student’s *t*-test. Significance is indicated as follows: *, *P* < 0.05; **, *P* < 0.01; ***, *P* < 0.001. Abbreviations: Fasting Glucose (GLU); Activated Partial Thromboplastin Time (Screening) (APTT-S); Activated Partial Thromboplastin Time (Ratio) (APTT-R); Prothrombin Time (Ratio) (PT-R); Prothrombin Time (Screening) (PT-S); Prothrombin Time Activity (PR%); Prothrombin Time - International Normalized Ratio (PT-INR).

(**B**). Heatmap displaying Spearman correlations between coagulation parameters and clinical characteristics of patients in Cohort 1. Significant correlations with an Benjamini-Hochberg (BH)-adjusted *P* value < 0.05 are marked with asterisks. Significance is indicated as follows: *, adjusted *P* < 0.05; **, adjusted *P* < 0.01; ***, adjusted *P* < 0.001.

(**C**). t-Distributed Stochastic Neighbor Embedding (t-SNE) plot for snRNA-seq data, with two groups: control (pink dots) and obese (green dots) (left). The t-SNE plot displays cell clusters based on 27,376 single-nucleus transcriptomes (right), with separation by groups.

Figure S2. Spatial transcriptomics deconvolution results.

(**A**). Spatial plot showing the AUC scores of Hep1-5 marker genes based on spatial transcriptomics of human normal liver.

(**B**). Dimplot of annotated spots categorized by central venous (CV), periportal (PP), and inter-zonal (IZ) regions using marker genes, with dimensionality reduction performed using Principal Component Analysis (PCA).

(**C**). Boxplots displaying the AUC scores of Hep1-5 marker genes for each spot within different zonal regions.

(**D, E**). Spatial plot showing the expression level of PP markers (*HAL*/*SDS*), CV markers (*GLUL*/*SLC1A2*) and fibrinogen genes (*FGA*/*FGB*/*FGG*) using healthy human liver spatial data (GSE243981, https://macparlandlab.shinyapps.io/healthylivermapspatialgui/).

(**F**). Dotplot displaying the expression level of PP markers (*HAL*/*SDS*), CV markers (*GLUL*/*SLC1A2*) across Hep1-Hep5 clusters.

(**G**). UMAP visualization comparing our hepatocyte clusters (Hep1-Hep5) with published zonation-defined clusters (GSE185477).

(**H**). GSEA analysis of Hep5 gene expression in obesity. Bar plot showing GSEA analysis based on KEGG category in Hep5 cells between obese and control individuals. The color of the bars indicates the normalized enrichment score (NES) value.

Figure S3. Validation of Coagulation-Related Hepatocyte Subpopulations Across Independent scRNA-Seq Datasets.

(**A**). UMAP plot showing six distinct hepatocyte subpopulations in healthy liver samples from GSE185477.

(**B**). Bubble plot of signature gene expression in the six hepatocyte clusters.

(**C**). Metascape analysis of enriched pathways among cluster 2 and cluster 4 signature genes.

(**D**). UMAP plot showing the distribution of four distinct hepatocyte clusters in cultured primary hepatocytes from healthy human liver in GSE180665.

(**E**). Bubble plot of *FGA*, *FGB* and *FGG* expression in cluster 2 hepatocytes.

(**F**). Metascape analysis of enriched pathways in signature genes of cluster 0-3.

Figure S4. Bulk RNA-seq deconvolution results.

(**A**). Column stacked plots of the proportions of 15 cell clusters in each sample in control (Con, N=12) and obese (Obe, N=19) groups deconvoluted by BisqueRNA and CIBERSORT, respectively.

(**B**). The proportions of 15 cell clusters deconvoluted by BisqueRNA and CIBERSORT from Con (N=12) and Obe (N=19) groups. Student’s *t*-test, *, *P* < 0.05; **, *P* < 0.01; ***, *P* < 0.001; ****, *P* < 0.0001.

(**C**). Heatmap showing the Spearman correlations of the proportions of 15 cell clusters (deconvoluted by BisqueRNA and CIBERSORT, respectively) and BMI of patients in Cohort 1. Significant correlations with BH-adjusted *P* value < 0.05 are labelled in the heatmap with asterisks. *, adjusted *P* < 0.05; **, adjusted *P* < 0.01; ***, adjusted *P* < 0.001.

Figure S5. Expression of fibrinogen transcripts in hepatocytes.

(**A**). Violin plots of fibrinogen transcript levels in the 5 hepatocyte clusters between control (Con, blue) and obese (Obe, red) individuals. Significant differences were determined by Wilcoxon test.

(**B**). Comparison of hepatic fibrinogen gene expression (*FGA*, *FGB*, *FGG*) and Hep1–5 cell proportions (estimated via BisqueRNA) between non-obese controls (Con, N=12), and obese patients stratified by MASLD severity: mild (NAS <5; Mild, N=9) vs. severe (NAS ≥5; Severe, N=10).

(**C**). Comparison of hepatic fibrinogen gene expression (*FGA*, *FGB*, *FGG*) and Hep1–5 cell proportions (estimated via BisqueRNA) between non-obese controls (Con, N=12), and obese patients stratified by lipid profiles: dyslipidemia (N=10) vs. normal (N=9). Transcriptomic differences were analyzed using DESeq2, with pairwise comparisons followed by multiple testing correction (Benjamini-Hochberg). Asterisks (*) denote statistically significant differences (adjusted *P* < 0.05).

Figure S6. Pseudotime trajectory of hepatocytes.

(**A**). Phylogenetic velocity fields reconstructed by PhyloVelo for the corresponding differentiation scenarios on the UMAP plot. Colors represent different clusters.

(**B**). UMAP plot of hepatocytes in the control (Con) and obese (Obe) groups labeled by pseudotime values with trajectories from Monocle3. Color gradient indicates pseudotime values. Transition-state cells differentiating from Hep1-4 to Hep5 are outlined in the grey dashed boxes, with a greater number of such cells observed in the Obe group.

(**C**). Gene bin plots showing the gene expression activities along the gene trajectory over the cell embedding. Genes along the trajectory are ordered and then split into seven equal-sized bins. The gene bin score is defined by the proportion of genes (from each bin) expressed in each cell. Arrows indicate the path of gene distribution progression over the cells.

(**D**). Expression profiles of the genes at the terminus of the trajectory.

Figure S7. Computational identification of CEBPB as a key regulator of IL6R expression in Hep5.

(**A**). UCSC Genome Browser view (hg38) of the IL6R gene locus (orange highlight) and its 2-kb upstream region, showing transcription factor binding site (TFBS) predictions from the JASPAR CORE vertebrate’s database (2022 release; minimum score threshold: 450). CEBPB binding sites (box) are prominently predicted immediately upstream of the IL6R transcription start site.

(**B**). JASPAR-based computational prediction of multiple high-confidence CEBPB binding motifs within the IL6R promoter region. Sequence logos indicate conserved nucleotide positions in predicted binding sites.

(**C**). Differential transcription factor (TF) activity analysis in Hep5 cells between obese (Obe) and control (Con) groups. The x-axis shows log_2_ fold-change (log_2_FC) of TF activity; point color represents statistical significance (-log_10_ adjusted *P*-value), while point size reflects the product of proportions (pt1×pt2) of cells with non-zero TF activity in both groups. CEBPB ranked as the second most significantly activated TF in Hep5.

Figure S8. CEBPB Regulates IL6-Induced Fibrinogen Expression in Hepatocytes.

Fibrinogen genes (*FGA*/*FGB*/*FGG*) mRNA levels in HepG2 cells treated with IL6 (10 ng mL^-1^) with or without CEBPB siRNA for 48 h. Data are presented as box plots showing the median and interquartile range, with individual data points shown (n = 3–5 replicates). Statistical significance was assessed using pairwise *t*-tests with Benjamini–Hochberg correction (**P*<0.05, ***P*<0.01, ****P*<0.001 vs. control; normalized to *ACTB*).

Figure S9. CEBPB knockdown reverses IL6-induced transcriptional reprogramming in primary human hepatocytes.

(A) Schematic of experimental design in primary human hepatocytes (PHHs): untreated control (NC), IL6 stimulation (10 ng mL^-1^), IL6 with CEBPB knockdown (IL6+siR-*CEBPB*), and CEBPB knockdown alone (siR-*CEBPB*) (n=3 biological replicates per group). Generated by *BioRender*.

(B) Principal component analysis (PCA) of global transcriptome profiles across treatment conditions.

(C) Volcano plot of differentially expressed genes (DEGs) in IL6 vs NC comparison (x-axis: log_2_ fold-change; y-axis: -log_10_ *P* value). Fibrinogen genes (*FGA*, *FGB*, *FGG*) and *CEBPB* are highlighted with gene labels.

(D) Gene Set Enrichment Analysis (GSEA) of KEGG pathways showing significant enrichment in IL6-treated vs control hepatocytes.

(E) KEGG pathway enrichment analysis of DEGs (IL6 vs NC), ranked by enrichment significance (-log_10_ *P* value).

(F) Volcano plot of DEGs in rescue condition (IL6+siR-*CEBPB* vs IL6 alone) (x-axis: log_2_ fold-change; y-axis: -log_10_ *P* value). Fibrinogen genes (*FGA*, *FGB*, *FGG*) and *CEBPB* are highlighted with gene labels.

(G) GSEA plot of KEGG pathways altered by *CEBPB* knockdown in IL6-stimulated hepatocytes.

(H) KEGG pathway enrichment of DEGs (IL6+siR-*CEBPB* vs IL6), showing pathways suppressed by CEBPB inhibition.

(I) Volcano plot of DEGs in CEBPB knockdown vs control (siR-*CEBPB* vs NC) (x-axis: log_2_ fold-change; y-axis: -log_10_ *P* value). Fibrinogen genes (*FGA*, *FGB*, *FGG*) and *CEBPB* are highlighted with gene labels.

(J) GSEA plot of KEGG pathways modulated by *CEBPB* knockdown under basal conditions.

(K) KEGG pathway enrichment of DEGs (siR-*CEBPB* vs NC), ranked by enrichment significance (-log_10_ *P* value).

(L) Log_2_FC values of hepatocyte pseudotime-associated genes across treatment comparisons (IL6 vs NC, IL6+ siR-*CEBPB* vs IL6, and siR-*CEBPB* vs NC) and their correlation coefficients with pseudotime values.

Figure S10. Helenalin acetate (HA)-mediated CEBPB inhibition attenuates IL6-dependent transcriptional rewiring in primary human hepatocytes.

(A) Schematic of experimental design in primary human hepatocytes (PHHs): untreated control (NC), IL6 stimulation (10 ng mL^-1^), IL6 stimulation (50 ng mL^-1^), and IL6 with helenalin acetate (HA, CEBPB inhibitor) (n=3 biological replicates per group). Generated by *BioRender*.

(B) Principal component analysis (PCA) of transcriptomes from primary human hepatocytes treated with: vehicle control (NC), IL6 (10 or 50 ng mL^-1^), or IL6 (10 ng mL^-1^) + HA (n=3 per group).

(C) Correlation analysis showing pseudotime-associated genes correlation coefficients with pseudotime trajectory are positively correlated with IL6-induced changes (log_2_FC IL6 vs NC) but negatively correlated with rescue conditions (log_2_FC IL6+HA vs IL6).

(D) Volcano plot of differentially expressed genes (DEGs) in IL6 (10 ng mL^-1^) vs NC comparison (x-axis: log_2_ fold-change; y-axis: -log_10_ *P* value). Fibrinogen genes (*FGA*, *FGB*, *FGG*) and *CEBPB* are highlighted with gene labels.

(E) Gene Set Enrichment Analysis (GSEA) of KEGG pathways showing significant enrichment in IL6-treated (10 ng mL^-1^) vs control hepatocytes.

(F) KEGG pathway enrichment analysis of DEGs (IL6 10 ng mL^-1^ vs NC), ranked by enrichment significance (-log_10_ *P* value).

(G) Volcano plot of differentially expressed genes (DEGs) in IL6 (50 ng mL^-1^) vs NC comparison (x-axis: log_2_ fold-change; y-axis: -log_10_ *P* value). Fibrinogen genes (*FGA*, *FGB*, *FGG*) and *CEBPB* are highlighted with gene labels.

(H) Gene Set Enrichment Analysis (GSEA) of KEGG pathways showing significant enrichment in IL6-treated (50 ng mL^-1^) vs control hepatocytes.

(I) KEGG pathway enrichment analysis of DEGs (IL6 50 ng mL^-1^ vs NC), ranked by enrichment significance (-log_10_ *P* value).

(J) Volcano plot of DEGs in rescue condition (IL6+HA vs IL6 10 ng mL^-1^ alone), demonstrating reversal of IL6 effects. Fibrinogen genes (*FGA*, *FGB*, *FGG*) and *CEBPB* are highlighted with gene labels.

(K) GSEA plot of KEGG pathways altered by *CEBPB* inhibition in IL6-stimulated hepatocytes.

(L) KEGG pathway enrichment of DEGs (IL6+HA vs IL6), ranked by enrichment significance (-log_10_ *P* value).

(M) Log_2_FC values of hepatocyte pseudotime-associated genes across treatment comparisons (IL6 10 ng mL^-1^ vs NC, IL6 50 ng mL^-1^ vs NC, and IL6+HA vs NC) and their correlation coefficients with pseudotime values.
